# Mixed‐Coordination Electrolytes With Molecular Additives for Robust Interphases in High‐Voltage Rechargeable Magnesium Batteries

**DOI:** 10.1002/advs.75749

**Published:** 2026-05-25

**Authors:** Dedy Setiawan, Toshihiko Mandai

**Affiliations:** ^1^ Functional Electrolyte Synthesis Team Research Center for Energy and Environmental Materials (GREEN) National Institute for Materials Science (NIMS) Tsukuba Ibaraki Japan

**Keywords:** anion coordination, electrolyte, interphase, magnesium batteries

## Abstract

Rechargeable magnesium batteries (RMBs) offer a low‐cost and high‐capacity alternative to the current energy storage systems, yet achieving high‐energy density and stable operation at high‐voltage remains difficult, particularly due to oxidative decomposition in conventional ether‐based electrolytes. In this work, we introduce a mixed‐coordination electrolyte (MCE) combined with a molecular additive (MCE‐MA) designed to promote anion‐derived interphases and enhance cycling stability in high‐voltage RMBs. The MCE formulation integrates dissociative and associative coordinating salts to optimize both Mg^2+^ transport and interphase formation, while the molecular additive facilitates the development of uniform, robust interphases. MCE‐MA exhibits improved Mg plating/stripping efficiency in Cu|Mg asymmetric cells and sustains over 250 h of stable cycling in Mg|Mg symmetric cells, significantly outperforming conventional halide‐free ether‐based electrolytes. Furthermore, MCE‐MA enables long‐term cycling of a full cell with oxide‐based cathode, achieving 200 cycles at 100 mA g^−1^. X‐ray photoelectron spectroscopy (XPS) and time‐of‐flight secondary ion mass spectrometry (ToF‐SIMS) were employed systematically to reveal the origin of enhanced performance.

## Graphical abstract

A mixed‐coordination electrolyte (MCE) combined with a molecular additive (MCE‐MA) promotes pseudo ion pair (pIP) formation within a solvent‐separated ion pair (SSIP)–rich environment, enabling a robust interphase that significantly improves the cycle performance of high‐voltage rechargeable magnesium batteries (RMBs).

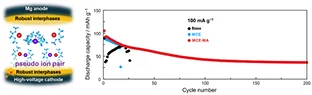

## 1 Introduction

The growing global reliance on lithium‐ion batteries (LIBs) has raised concerns over the long‐term availability and cost of lithium resources [1]. This has stimulated the search for alternative energy‐storage systems based on earth‐abundant elements. Among these, rechargeable magnesium batteries (RMBs) are promising candidates owing to the low cost and natural abundance of magnesium, combined with its high volumetric and gravimetric capacities (3833 mAh cm^−2^ and 2205 mAh g^−1^, respectively). When coupled with high‐energy‐density cathodes, RMBs could, in principle, deliver energy densities comparable to state‐of‐the‐art LIBs [2].

Despite significant progress, the practical realization of high‐energy RMBs remains hindered by long‐standing challenges in electrolyte design, particularly when operated at high‐voltage [3, 4, 5]. Electrolytes based on weakly coordinating anion salts (WCA) such as Mg[B(hfip)_4_]_2_ and Mg[Al(hfip)_4_]_2_ (hfip:hexafluoroisopropanol) in ether solvents exhibit excellent reductive stability and highly reversible Mg plating/stripping (>99% efficiency for 0.3 m Mg[Al(hfip)_4_]_2_ in diglyme), owing to weak cation–anion interactions that suppress surface passivation on the Mg anode [6, 7, 8, 9, 10]. However, the cycle performance of high‐voltage RMBs with this electrolyte system remains poor, which is mainly attributed to the high fraction of uncoordinated ether molecules that are readily exposed at the cathode interface and undergo oxidative decomposition at high voltage [3, 11, 12, 13]. As a result, issues related to solvent‐derived decomposition, active proton formation, interphase instability, and cathode component dissolution were recently discovered, and are also commonly found in the current LIBs research [3, 4, 14, 15, 16]. Although employing high‐concentration electrolytes has shown some promise in LIBs as well as RMBs, the relatively high cost of some Mg salts makes this approach suboptimal [17, 18].

**FIGURE 1:**
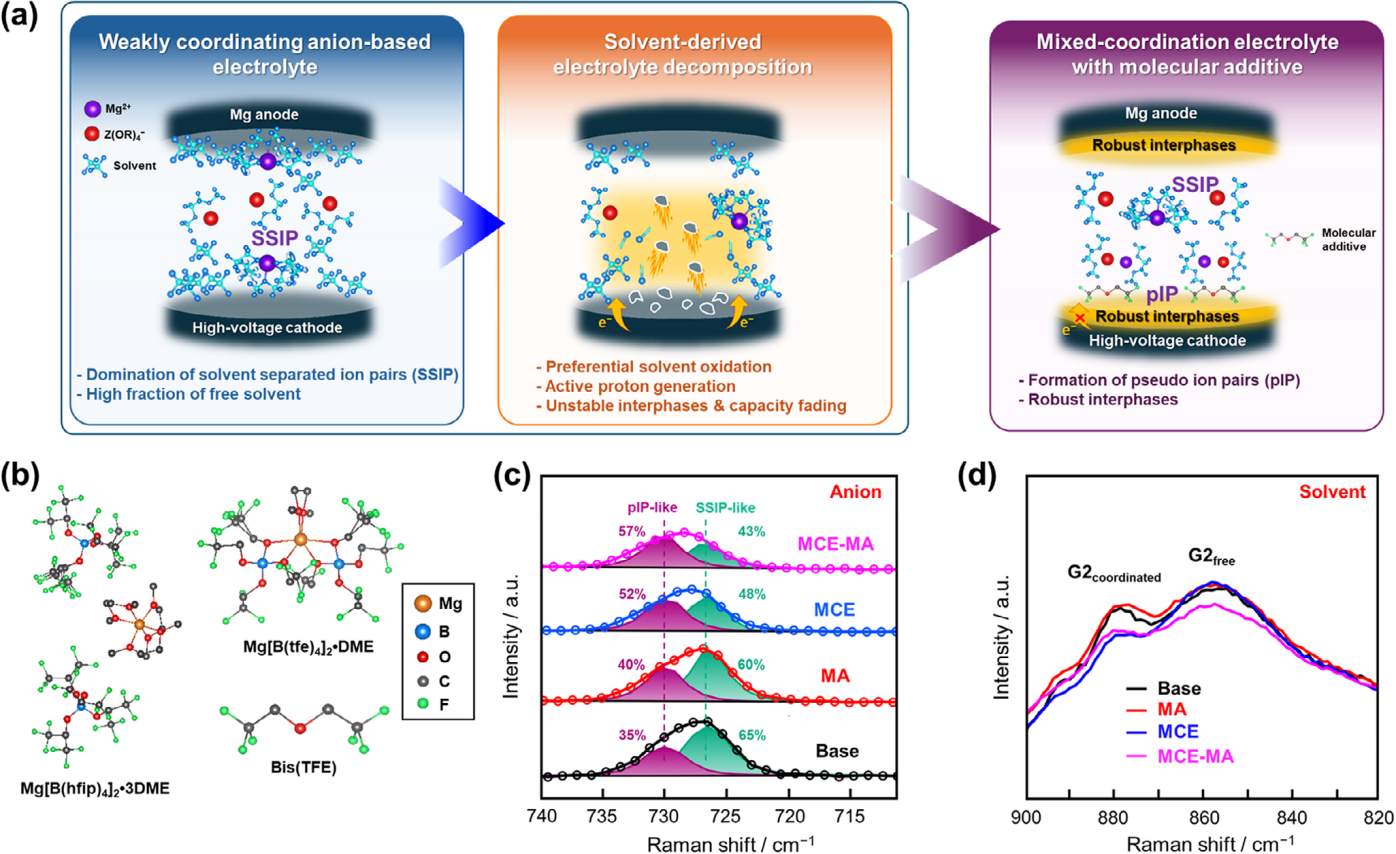
(a) Schematic illustration of issues in WCA‐based electrolyte and the introduction of MCE‐MA to enable robust interphases formation. (b) Molecular structure of Mg[B(hfip)_4_]_2_•3DME salt, Mg[B(tfe)_4_]_2_•DME salt, and BisTFE molecule. Hydrogen and disordered atoms are not shown for figure clarity. (c) Raman spectra of the as‐prepared electrolytes in the anion‐sensitive spectral region, together with peak deconvolution analysis. The percentages indicate the relative contributions of solvent‐separated ion pair (SSIP) and pseudo ion pair (pIP) in each electrolyte. (d) Raman spectra of as‐prepared electrolytes in the selected region sensitive to solvent with assigned free and coordinated diglyme (G2).

In this study, we introduce a WCA‐based electrolyte incorporating mixed anionic species and a molecular additive to promote stable interphase formation for extended cycle performance of high‐voltage RMBs. Specifically, Mg[B(hfip)_4_]_2_ in diglyme (G2) was used as the base electrolyte, while a certain concentration of Mg[B(tfe)_4_]_2_ salt (tfe:trifluoroethanol) was added to modulate the solvation structure and help the formation of anion‐derived interphase, forming a mixed coordination electrolyte (MCE). Additionally, Bis(2,2,2‐trifluoroethyl) ether (BisTFE) was chosen as a molecular additive to promote homogeneous and robust interphase. The resulting mixture, termed mixed‐coordination electrolyte with molecular additive (MCE‐MA), is summarized in Figure 1a.

Although both Mg[B(hfip)_4_]_2_ and Mg[B(tfe)_4_]_2_ salts contain bulky weakly coordinating anions, their cation–anion interactions differ significantly. In Mg[B(hfip)_4_]_2_, the coordination structure is fully dissociative, where Mg^2+^–glyme complexes are separated from [B(hfip)]^−^ anions (Figure 1b) [6]. The strong electron‐withdrawing hfip groups suppress anion coordination, while the strong electric field of Mg^2+^ enhances glyme solvation, resulting in a glyme‐dominated solvation shell [12]. In contrast, Mg[B(tfe)_4_]_2_ salt has an associative coordination structure (Figure 1b), where Mg^2+^ binds to six oxygen atoms from two [B(tfe)_4_]^−^ anions and one glyme molecule. This stronger cation–anion association arises from the weaker charge dispersion of [B(tfe)_4_]^−^, as the tfe group contains only one ─CF_3_ group compared to two in the hfip group [19]. Consequently, Mg[B(tfe)_4_]_2_ coordinates fewer glyme molecules (one vs. three in Mg[B(hfip)_4_]_2_). The combination of these two salts is expected to balance efficient Mg^2+^ transport with anion‐derived interphase formation, while the addition of BisTFE, which has a linear fluorinated structure (Figure 1b), further promotes a uniform interphase.

## 2 Results and Discussion

### 2.1 Electrolyte Solvation Structure

The synthesis of Mg salts and the preparation of electrolytes are detailed in the Experimental Section. Both Mg[B(hfip)_4_]_2_ and Mg[B(tfe)_4_]_2_ form adducts with coordinated DME (dimethoxyethane) used during the synthesis, as confirmed by ^1^H NMR (Figure S1), consistent with previous reports [9, 19]. The base electrolyte was prepared using 0.3 m Mg[B(hfip)_4_]_2_ in diglyme (G2), a concentration selected based on its previously reported electrochemical performance [9]. To formulate the MCE, 5 mM Mg[B(tfe)_4_]_2_ was included in the base electrolyte, while the MCE‐MA was obtained by introducing 1 vol% BisTFE into the MCE. The detailed electrolyte compositions are summarized in Table S1. The concentration of Mg[B(tfe)_4_]_2_ and BisTFE was chosen after initial screening with a symmetric Mg|Mg cell and a full‐cell test. 5 mM Mg[B(tfe)_4_]_2_ and 1 vol% BisTFE provide balanced performance in both electrochemical tests.

To investigate the influence of mixing WCA salts with a distinct coordination behavior, as well as the effect of a molecular additive, Raman spectra were collected at room temperature for the base electrolyte, base + BisTFE (MA), MCE, and MCE‐MA. The regions sensitive to anion and solvent coordination are shown in Figure 1c,d, respectively. In the 720–740 cm^−1^ region (Figure 1c), corresponding to anion coordination, MCE and MCE‐MA exhibit a blueshift relative to the base electrolyte, indicating the formation of more “pseudo ion‐pair” clusters, despite a minimum amount of Mg[B(tfe)_4_]_2_ being incorporated (only 5 mM) [20, 21, 22]. According to the peak deconvolution (Figure 1c), the base electrolyte is dominated by solvent‐separated ion pairs (SSIP), with partial contribution from pseudo ion pair (pIP). Here, the term “pseudo ion pair (pIP)” is used to describe a weak cation–anion association characteristic of WCA‐based electrolytes, in which the anion resides in close proximity to Mg^2+^ without forming a classical inner‐sphere contact ion pair (CIP). Although Mg[B(hfip)_4_]_2_ is widely regarded as prototypical WCA‐salt, recent studies have reported and discussed the presence of such cation‐anion association [10, 23]. Notably, in the MCE and MCE‐MA, the contribution of this cation‐anion association becomes more dominant (Figure 1c). As Mg[B(tfe)_4_]_2_ alone is known to favor pIP in ethers, introducing even a small fraction of this salt into the base electrolyte significantly alters the equilibrium between cation–solvent and cation–anion interactions. The [B(tfe)_4_]^−^ anion possesses a higher local charge density and weaker electron‐withdrawing ability compared to [B(hfip)_4_]^−^, making it more nucleophilic toward Mg^2+^. As a result, it can outcompete glyme for coordination sites, promoting the formation of pIP and partially disrupting the dissociative solvation structure characteristic of the base electrolyte. This enhanced anion participation reduces the availability of free G2 molecules and weakens Mg^2+^–G2 interactions.

In the 820–900 cm^−1^ region (Figure 1d), associated with solvent coordination, the coordinated G2 peak intensity is reduced in MCE and MCE‐MA compared to the base electrolyte. This suggests a weakening of Mg^2+^–G2 interactions due to increased anion participation in the pIP formation, in good agreement with the anion‐sensitive region peaks [24]. In the MCE‐MA, the further decrease in free G2 intensity likely arises from weak but non‐negligible interactions between Mg^2^^+^ and the BisTFE molecule. While BisTFE is generally non‐coordinating, its electron‐withdrawing CF_3_ groups can polarize the surrounding solvation shell, subtly stabilizing Mg^2+^–anion complexes and homogenizing the overall solvation environment [25, 26, 27]. In contrast, addition of BisTFE alone to the base electrolyte does not perturb the solvation structure substantially because, without the associative anion [B(tfe)_4_]^−^, there is no competitive driving force to reorganize the coordination equilibrium.

### 2.2 Anion‐Derived Solid Electrolyte Interphase

**FIGURE 2:**
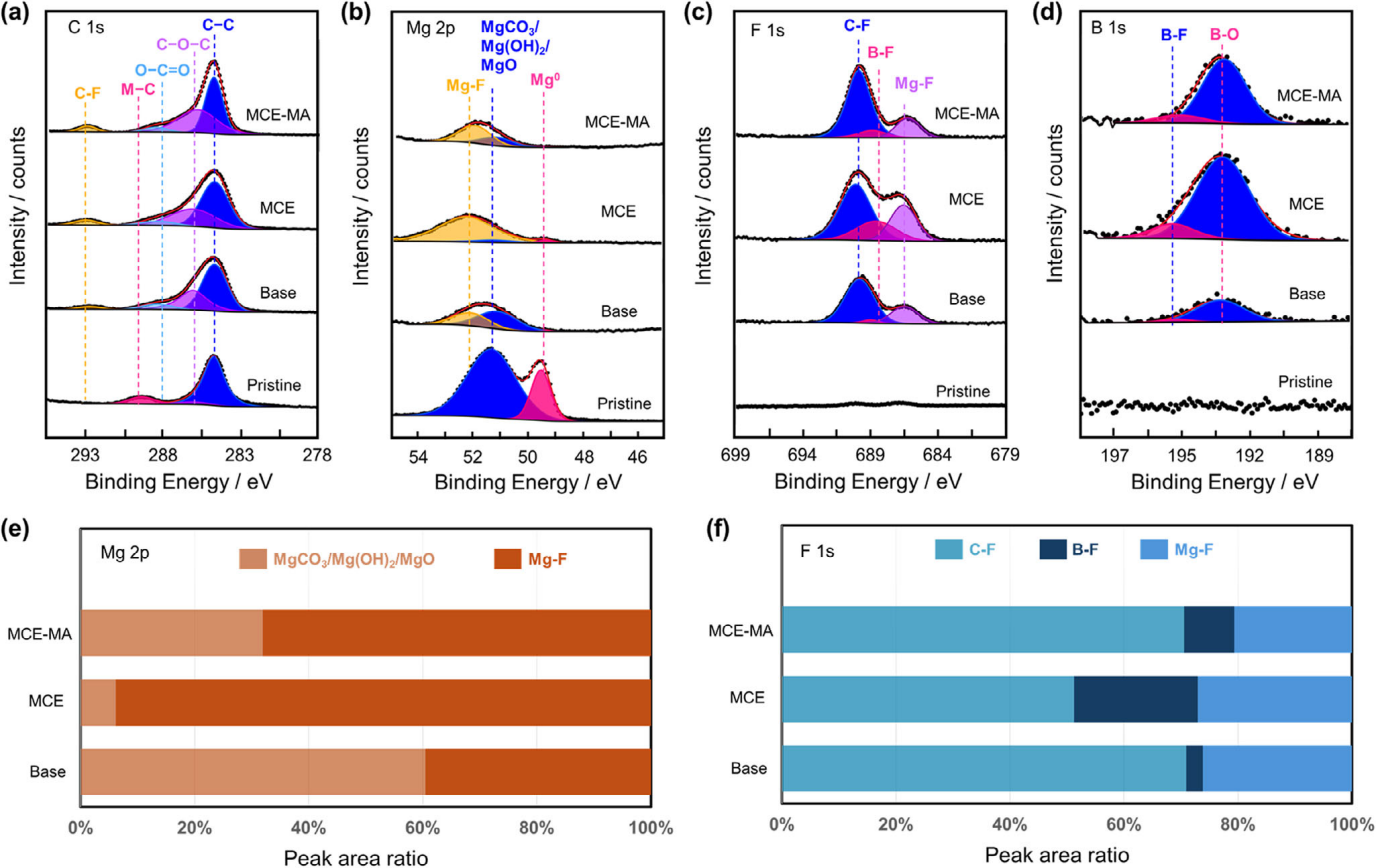
Ex situ XPS survey spectra of Mg metal cycled in Mg|Mg symmetric cell (0.5 mA cm^−2^, 0.25 mAh cm^−2^) for 50 h using base electrolyte, MCE, and MCE‐MA; (a) C 1s, (b) Mg 2p, (c) F 1s, and (d) B 1s. (e,f) The fitted peak area ratio of (e) Mg 2p and (f) F 1s provides quantitative results of XPS survey spectra.

Modulation of the Mg^2+^ solvation environment is ultimately manifested in the chemical composition of the solid electrolyte interphase (SEI) formed on Mg metal. In particular, the formation of pIP‐rich coordination structures in the MCE and MCE‐MA electrolytes is expected to shift the initial interfacial reactions from solvent‐dominated decomposition toward preferential anion participation. To elucidate how these solvation changes translate into interphase chemistry, ex situ X‐ray photoelectron spectroscopy (XPS) was performed on Mg metal electrodes recovered after 50 h cycled in a Mg|Mg symmetric cell at 0.5 mA cm^−2^ and 0.25 mAh cm^−2^ (Figure S2a) using the three electrolytes (Figure 2a–d).

The C 1s spectra (Figure 2a) reveal the presence of organic surface species and fluorinated carbonaceous components (C─F) for all electrolytes. Notably, MCE‐MA exhibits the highest C─F peak intensity, indicating enhanced formation of fluorocarbons and related species, which are attributed to the controlled decomposition of fluorinated anions and/or the BisTFE additive. This observation already suggests a transition toward an anion‐derived SEI chemistry in the mixed‐coordination systems. Consistent with this trend, the Mg 2p spectra (Figure 2b) show contributions from Mg─F species (≈52.0 eV) coexisting with Mg─O–related components arising from oxides, hydroxides, and/or carbonates (≈50.9 eV) [28]. Both MCE and MCE‐MA display a substantially higher Mg─F fraction than the base electrolyte, further evidencing a stronger anion‐derived interphase. Interestingly, a small metallic Mg^0^ signal is detected in the MCE sample but is absent in MCE‐MA, implying that the SEI formed in MCE‐MA is more homogeneous and likely provides more complete surface coverage. The F 1s spectra (Figure 2c) provide direct insight into fluorine‐containing interphase species. A dominant peak at ∼689 eV corresponds to C─F bonds and is most pronounced in MCE‐MA, in agreement with the C 1s analysis [29]. In addition, a shoulder at ∼685 eV assigned to Mg─F is observed in all electrolytes but is significantly intensified in MCE, suggesting substantial anion‐derived inorganic fluoride formation. Given the boron‐centered anions employed, a B─F contribution near ∼688 eV was also included in the peak fitting. The enhanced B─F intensity observed for both MCE and MCE‐MA relative to the base electrolyte further confirms the active involvement of anion decomposition in shaping the SEI [30, 31]. This conclusion is reinforced by the B 1s spectra (Figure 2d), which show markedly stronger B─O (∼193 eV) and B─F (∼195 eV) signals for MCE and MCE‐MA than for the base electrolyte, consistent with increased boron‐containing decomposition products within the interphase [32].

To provide a more quantitative comparison, the fitted peak area ratios of the Mg 2p and F 1s spectra are summarized in Figure 2e,f. As shown in Figure 2e, Mg─F species become the dominant Mg‐containing component in both MCE (94%) and MCE‐MA (68%), in sharp contrast to the base electrolyte (39%). Furthermore, the fluorinated interphase composition (Figure 2f) is dominated by C─F species across all samples: base electrolyte (71%), MCE (51%), and MCE‐MA (71%). On the other hand, B─F contributions are significantly more pronounced in the mixed‐coordination electrolytes, MCE (22%) and MCE‐MA (9%), reflecting enhanced and controlled anion participation during SEI formation. Overall, both MCE and MCE‐MA promote the formation of anion‐derived SEI layers; however, MCE‐MA likely has a more robust and homogeneous SEI structure, due to the incorporation of BisTFE additive.

BisTFE is expected to exhibit preferential reductive and/or oxidative decomposition at the electrode interface due to its relatively labile C─O bonds and electron‐withdrawing ─CF3 groups. Such decomposition can generate fluorinated species (e.g., C─F─containing fragments), which contribute to the formation of a fluorine‐rich interphase. This is consistent with the enhanced C─F signals observed in the XPS (C 1s and F 1s) spectra, where fluorinated fragments are more uniformly distributed in the MCE‐MA system. In addition to contributing fluorinated species, the presence of BisTFE is also considered to modulate interfacial reactions by altering the local solvation environment near the electrode surface. This can lead to more homogeneous decomposition pathways, suppressing localized solvent degradation and promoting the formation of a more uniform and stable interphase layer [33, 34].

### 2.3 Impact of SEI on Mg Plating/Stripping Performance

**FIGURE 3:**
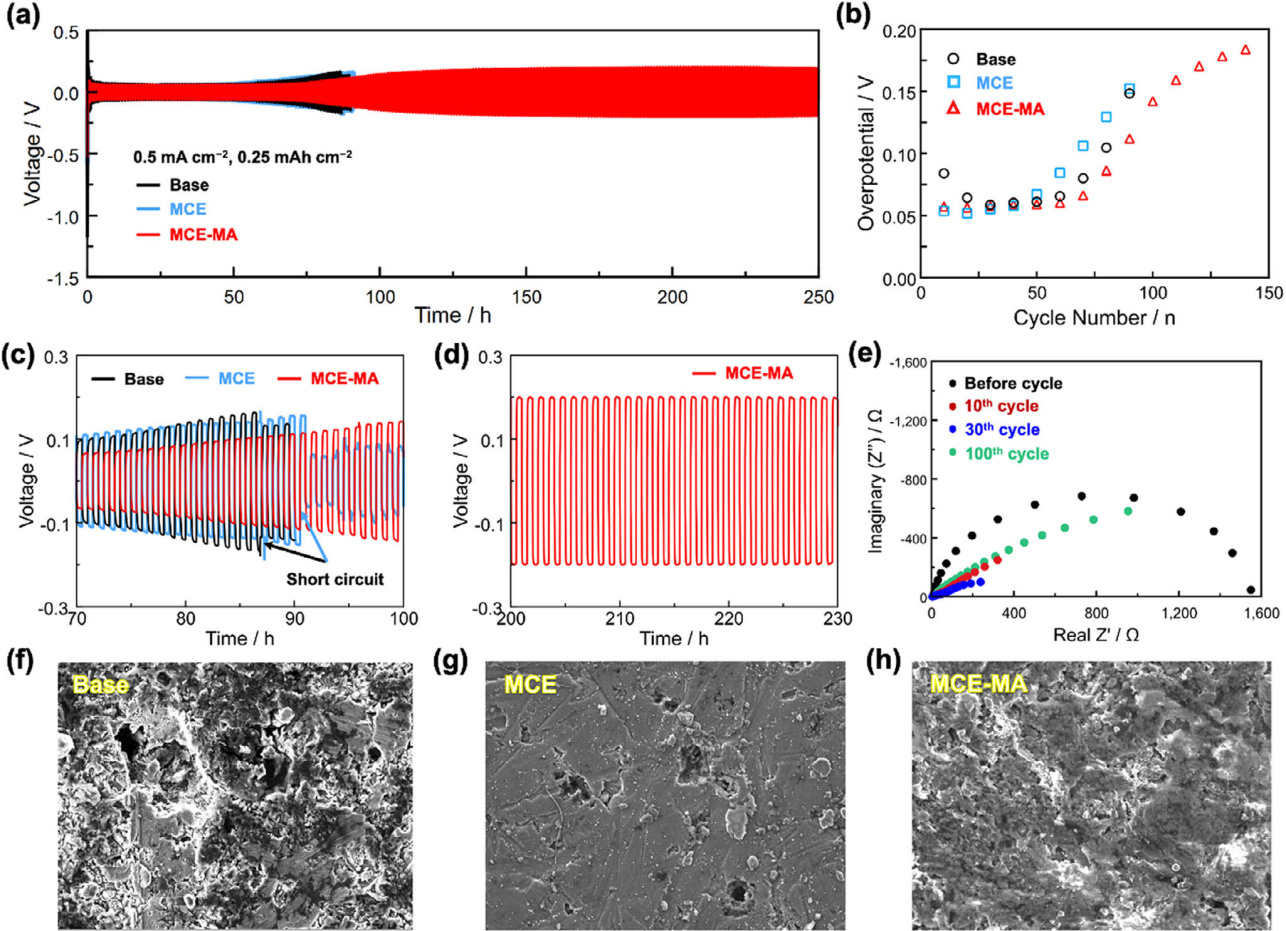
(a) The discharge‐charge profile of symmetric Mg|Mg cells with base electrolyte, MCE, and MCE‐MA, cycled at 0.5 mA cm^−2^ and 0.25 mAh cm^−2^. (b) Evolution of Mg plating/stripping overpotential as a function of cycle number in the symmetric cell. The overpotential is determined as the voltage plateaus of Mg plating/stripping at the end of the cycling test. (c,d) The magnified discharge/charge profile of the symmetric cell at (c) intermediate cycles, (d) later cycles. (e) Nyquist plot of EIS measurement of symmetric Mg|Mg cell with MCE‐MA electrolyte taken at different stages: before cycle, 10th cycle, 30th cycle, and 100th cycle. (f–h) SEM image of Mg metal surface after cycling in (f) base electrolyte, (g) MCE, and (h) MCE‐MA.

To evaluate the impact of SEI composition on the long‐term Mg plating/stripping stability, symmetric Mg|Mg cells were further tested in the three electrolytes at 0.5 mA cm^−2^ and 0.25 mAh cm^−2^ for longer hours. For the base electrolyte, repeated polarization (activation process) is required to initiate Mg plating/stripping (Figure S2b), reflecting the highly insulating nature of the Mg metal surface, as also observed previously [35]. In contrast, such behavior is absent in the MCE and MCE‐MA electrolytes, suggesting more favorable initial interfacial conditions for Mg electrochemistry. Despite these differences, all electrolytes exhibit comparable voltage profiles during the initial cycling period (Figure 3a), indicating similar interfacial kinetics at the early stage. However, pronounced differences emerge upon prolonged operation. Notably, the MCE‐MA electrolyte sustains stable Mg plating/stripping for more than 250 h, whereas both the base electrolyte and MCE suffer from soft short‐circuiting within approximately 100 h. It is worth noting that the Mg plating/stripping stability in the base electrolyte and the MCE is nearly identical. This observation indicates that the MCE alone does not significantly improve Mg plating/stripping homogeneity, despite the formation of an anion‐derived interphase, likely due to the inhomogeneous nature of the resulting interphase. In contrast, the incorporation of the molecular additive in MCE‐MA promotes a more homogeneous interphase, thereby enabling markedly enhanced and prolonged Mg plating/stripping stability.

The evolution of Mg plating/stripping overpotential provides further insight into the origin of these stability differences. As summarized in Figure 3b, all three electrolytes display similarly low overpotentials (<0.1 V) during the initial cycling stage (<50 cycles). Upon extended cycling, the overpotentials of the base electrolyte and MCE gradually increase from ∼0.1 to ∼0.15 V after ∼90 cycles, followed by a sudden drop in polarization associated with soft short‐circuit behavior (Figure 3c). Such abrupt voltage collapse is characteristic of internal shorting caused by non‐uniform Mg deposition [36, 37]. In contrast, although the overpotential of MCE‐MA also increases to ∼0.15 V after ∼100 cycles, no sudden voltage drop is observed. Instead, MCE‐MA maintains a stable overpotential of ∼0.2 V over more than 200 cycles (Figure 3d), demonstrating markedly enhanced durability. It is noteworthy that the Mg plating/stripping overpotential in all three electrolytes exhibits only a weak dependence on current density over the range of 0.25–2.0 mA cm^−2^, indicating that the use of MCE and MCE‐MA does not substantially alter the intrinsic Mg plating/stripping kinetics. In contrast, all cells fail when subjected to a high current density of 4.0 mA cm^−2^ (Figure S3), suggesting a common limitation under aggressive operating conditions rather than electrolyte‐specific kinetic effects.

The Nyquist plots (Figure 3e) from electrochemical impedance spectroscopy (EIS) measurement of a symmetric Mg|Mg cell with MCE‐MA reveal a clear evolution of the interfacial resistance during cycling. The initial impedance before cycling is relatively high, which can be attributed to the absence of a well‐formed interphase. After 10 cycles, the interfacial resistance decreases significantly, indicating the formation of an electrochemically active and ion‐conductive interphase. Upon further cycling (30th and 100th cycles), the impedance gradually increases, which is consistent with the growth and stabilization of the interphase layer during prolonged operation. Importantly, despite this increase at later stages, the overall impedance remains stable without an abrupt rise, in agreement with the sustained electrochemical performance observed for the MCE‐MA electrolyte. This behavior supports the formation of a robust and stable interphase that effectively regulates Mg plating/stripping over extended cycling.

We also observed that these differences in electrochemical stability are closely correlated with the morphology of Mg metal after cycling. SEM images of Mg electrodes retrieved from the symmetric cells are shown in Figure 3f–h. After cycling in the base electrolyte, the Mg surface becomes highly mossy and contains pronounced localized pits, indicative of uneven, 3D Mg deposition (Figure 3f). In the case of MCE, the surface is generally smoother; however, distinct localized holes remain, suggesting spatially heterogeneous Mg plating/stripping arising from a non‐uniform SEI (Figure 3g). By contrast, Mg cycled in MCE‐MA exhibits a relatively compact and homogeneous surface morphology, with significantly suppressed pit formation (Figure 3h). Overall, the electrochemical and morphological results indicate that the superior long‐term Mg plating/stripping stability in MCE‐MA originates from the formation of a more homogeneous and robust SEI. This stabilized interphase effectively mitigates localized current concentration and suppresses non‐uniform Mg growth, thereby preventing premature short‐circuiting during extended cycling.

Apart from Mg plating/stripping stability, we also discovered that the three electrolytes exhibited different plating/stripping efficiency trends. The Mg plating/stripping efficiency in the base electrolyte, MCE, and MCE‐MA was examined in asymmetric Cu|Mg cells at a current density of 1 mA cm^−2^ and areal capacity of 1 mAh cm^−2^. All three electrolytes exhibited comparable short‐term stability, with short‐circuiting observed after approximately 25 h (Figure S4a). However, the initial Mg nucleation overpotential revealed different behavior between the electrolytes (Figure S4c). The MCE and MCE‐MA display lower Mg nucleation overpotential compared to the base electrolyte, indicating more favorable Mg nucleation and improved interfacial reversibility. Besides, their Coulombic efficiency (CE) profiles revealed distinct trends. In the initial cycle, MCE showed the highest CE (96.67%) and the lowest overpotential, outperforming the base (89.55%) and MCE‐MA (93.13%). By the 14th cycle (before the short‐circuit), however, MCE‐MA surpassed both systems, achieving a CE of 94.84% with the lowest overpotential, while the base and MCE stabilized at 92.28% and 91.30%, respectively. These results highlight the plausible differences in SEI evolution among the electrolytes, driven by variations in their solvation structures. In MCE‐MA, SEI formation proceeds more gradually but yields a robust and stable interphase after repeated cycling. Conversely, MCE promotes rapid SEI formation during the initial cycles; however, the resulting interphase appears less resilient, leading to performance degradation over time.

Beyond SEI chemistry, the electrolyte composition also exerts a strong influence on Mg deposition behavior, as reflected by differences in the nucleation overpotential (Figure S5). To examine the resulting Mg plating morphology, Mg was electrodeposited onto Cu foil at a current density of 0.25 mA cm^−2^ with an areal capacity of 1.0 mAh cm^−2^, followed by SEM analysis. The corresponding morphologies for the base electrolyte, MCE, and MCE‐MA are shown in Figure S6. In the base electrolyte, Mg deposits consist of small, sparsely distributed nuclei, indicative of a high nucleation barrier. In contrast, Mg deposited from the MCE and MCE‐MA electrolytes exhibits larger feature sizes and a more compact, uniform morphology. This trend is consistent with previously reported correlations between reduced nucleation overpotential and increased metal deposit size [38, 39]. Such compact Mg deposition is advantageous for minimizing localized current concentration and suppressing undesired anode–electrolyte side reactions during repeated charge–discharge cycling.

Additionally, XRD analysis was also conducted for the Mg deposit obtained from the base electrolyte and MCE‐MA. The diffraction patterns reveal a noticeable change in preferred orientation (Figure S14): the (002) and (101) reflections become slightly more intense relative to the (100) peak when MCE–MA is used. This suggests that MCE–MA promotes crystal growth along the *c*‐axis of the hexagonal close‐packed Mg lattice. Such behavior can be attributed to additive‐induced modifications of the electrode surface energy and the adsorption environment at the metal–electrolyte interface, which can selectively stabilize certain crystallographic planes during nucleation and growth [40]. However, the detailed mechanism of this preferred (002) and (101) crystal growth requires further investigation and is beyond the scope of this study.

### 2.4 Insight Into High‐Voltage RMBs Application

**FIGURE 4:**
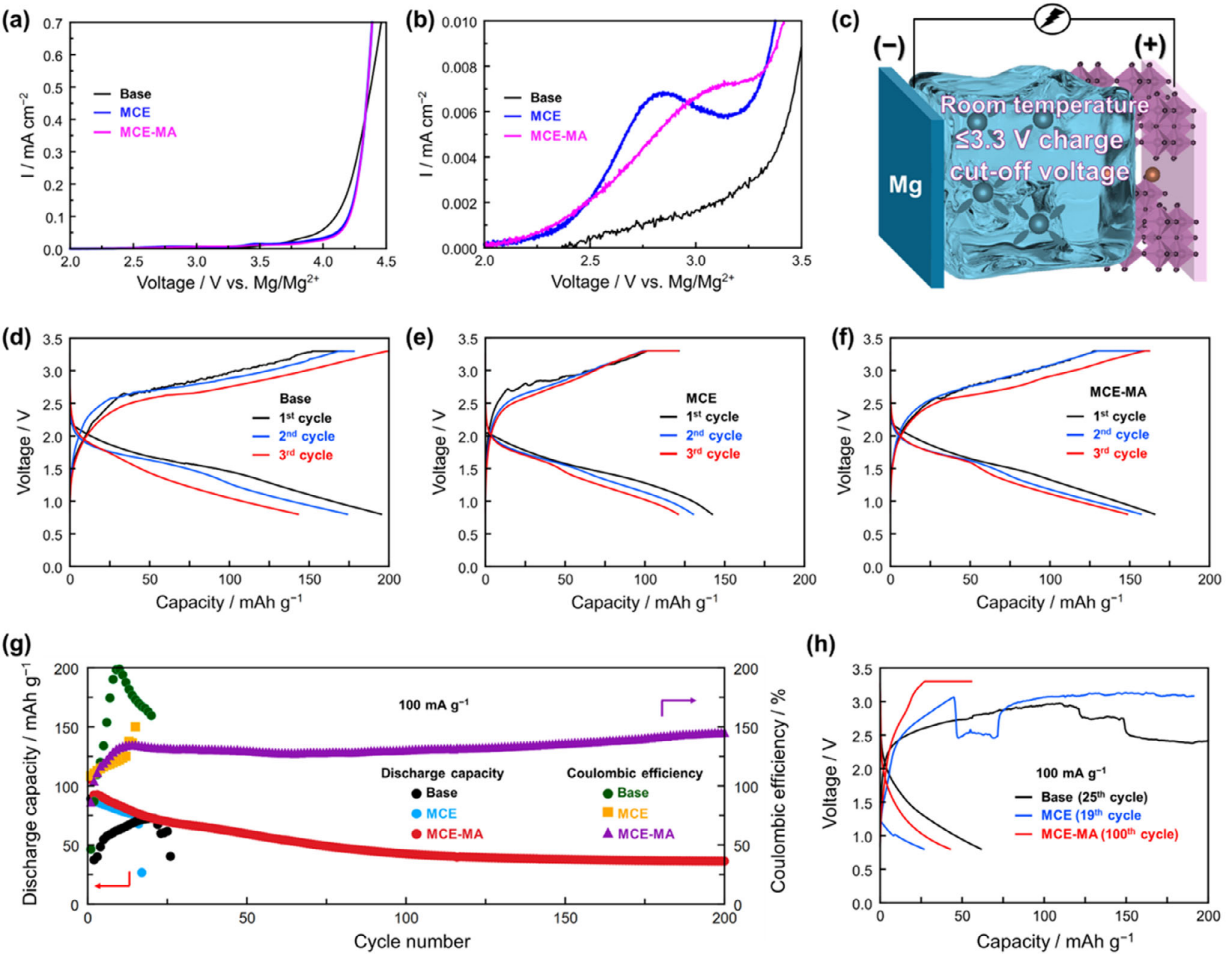
(a) LSV profile of the base electrolyte, MCE, and MCE‐MA measured using a Pt working electrode. (b) Magnified view of the LSV profile over the selected high voltage region. (c) Schematic illustration of high‐voltage RMBs comprising base electrolyte, MCE, or MCE‐MA with certain condition requirements. (d–f) Three representative cycles of galvanostatic discharge‐charge profile of RMBs comprising FeVO cathode with (d) base electrolyte, (e) MCE, (f) MCE‐MA at 10 mA g^−1^. (g) Cycling performance of RMBs with FeVO cathode in different electrolyte systems at 100 mA g^−1^ after 1 cycle conditioning at 10 mA g^−1^. The Coulombic efficiency was defined as Q_charge_/Q_discharge_ × 100%. (h) Selected discharge‐charge profiles highlighting the failure of the RMBs with base electrolyte and MCE.

To evaluate the applicability of the developed electrolyte systems for high‐voltage RMBs, linear sweep voltammetry (LSV) was performed first to provide insight into their oxidative stabilities. Figure 4a shows the LSV profiles with platinum (Pt) as the working electrode. Both MCE and MCE‐MA exhibit slightly enhanced oxidation stability compared with the base electrolyte. This improvement can be attributed to thermodynamic stabilization arising from the modified solvation structure in the mixed‐anion environment. In MCE and MCE‐MA, the coexistence of [B(hfip)_4_]^−^ and [B(tfe)_4_] ^−^ anions—along with the weakly coordinating BisTFE molecule in MCE‐MA—modulates the local coordination sphere around Mg^2+^. This mixed coordination weakens the strong Mg^2+^–G2 interactions dominant in the base electrolyte, leading to a more delocalized charge distribution and improved oxidative stability [41].

As shown in Figure 4b, small oxidation features appear below 3.3 V vs. Mg/Mg^2+^ for MCE and MCE‐MA, which are attributed to localized, self‐limiting cathode electrolyte interphase (CEI) formation. The mixed‐anion formulation slightly raises the bulk oxidative stability; however, local interfacial heterogeneities—such as regions enriched in [B(tfe)_4_]^−^ or BisTFE and high‐field sites on the electrode—can still host species with higher highest occupied molecular orbital (HOMO) energies that oxidize preferentially. Oxidation of these localized species, likely involving [B(tfe)_4_]^−^ or BisTFE, generates reactive radicals that decompose into boron‐ and fluorine‐containing CEI components. The limited charge associated with these peaks and the rapid current decay indicate the formation of a thin, passivating interphase that suppresses further oxidation.

While LSV provides a comparative assessment of the oxidative stability of the electrolytes, it primarily reflects dynamic electrochemical behavior. Therefore, the long‐term stability of the cathode–electrolyte interphase (CEI) is more reliably evaluated through galvanostatic full‐cell cycling with a cathode material at a reliable test condition. In this regard, the consistency between the LSV results and the extended cycling performance of the full cell should support the enhanced oxidative stability and interphase robustness of the MCE‐MA electrolyte.

Considering these oxidative stability results, two key conditions were established for assembling RMBs full cells: (1) operation at room temperature, due to the relatively low boiling point of BisTFE (62°C–63°C); and (2) a charge cut‐off voltage around 3.3 V to minimize parasitic electrolyte decomposition (Figure 4c). Based on these criteria, FeV_3_O_9_·1.1H_2_O (FeVO) was selected as a model cathode material because of its stable operation at the desired voltage range [42, 43]. Additionally, the intrinsic stability of molecular H_2_O molecules in FeVO has been systematically verified in previous studies, ensuring safe operation when paired with Mg metal anodes [43, 44]. The FeVO cathode was synthesized following earlier protocol, and was confirmed by X‐ray diffraction (XRD) and SEM–EDX analyses (Figure S7) [43].

Figure 4d–f presents the charge–discharge profiles of RMBs full cells with FeVO cathode at 10 mA g^−1^ using base electrolyte, MCE, and MCE‐MA. The applied current corresponds to an areal current density of approximately 0.01 mA cm^−2^ for the Mg anode. Despite identical testing conditions, clear performance differences are observed among the electrolytes. The cell with base electrolyte delivers the highest first discharge capacity of 197 mAh g^−1^ but suffers from rapid capacity fading to 150 mAh g^−1^ (76% of the first discharge capacity) after three cycles. The cell with MCE delivers the lowest first discharge capacity of 142 mAh g^−1^ and degrades to 121 mAh g^−1^ (85% of the first discharge capacity) after three cycles. The cell with MCE‐MA delivers a first discharge capacity of 166 mAh g^−1^ and has relatively more stable cycling behavior compared to the base electrolyte and MCE, with the third discharge capacity of 149 mAh g^−1^ (90% of the first discharge capacity). To elucidate the origin of the capacity discrepancy between different electrolyte systems, ICP–MS analysis was performed to quantify Mg content in the FeVO cathode after discharge (Figure S8). The fraction of discharge capacity attributed to Mg intercalation differs across electrolytes: 41% for the base electrolyte, 65% for MCE, and 64% for MCE‐MA. These results indicate that parasitic side reactions, likely proton insertion, are more pronounced in the base electrolyte, despite the low water content. As reported previously, such reactions occur during both discharge and charge, consistent with the higher charge capacity relative to discharge capacity observed after the second and third cycles in the base electrolyte [3]. Solvent‐derived decomposition not only reduces efficiency but also leads to interphase crossover, disrupting the uniformity of Mg plating/stripping at the anode and thereby compromising long‐term cycling stability [3]

Since MCE and MCE‐MA demonstrated the formation of anion‐derived interphases in the Mg anode, the impact on the RMBs full cell should be investigated. The cycling performance of the RMB full cell with the FeVO cathode was further evaluated at a higher current density of 100 mA g^−1^. Direct application of such high current densities led to poor cell performance for all electrolytes (not shown here), likely due to the insufficiently developed interphase discussed earlier. To address this, the full cells were preconditioned at a lower current density (10 mA g^−1^) for one formation cycle before subsequent cycling at 100 mA g^−1^ (Figure 4g).

Under these optimized conditions, MCE‐MA exhibited markedly superior long‐term stability, sustaining over 200 cycles with capacity retention of 40% after 100 cycles and 35% after 200 cycles (Figure 4g). In sharp contrast, cells employing the base or MCE electrolytes became inoperable within 30 cycles. Representative charge–discharge profiles of the base and MCE cells prior to failure are shown in Figure 4h. Both displayed elongated and non‐linear charge curves below the cut‐off voltage, suggesting the occurrence of abnormal electrochemical processes. Such behavior could stem from multiple degradation pathways, including soft short‐circuiting caused by electrode component migration or possible corrosion of the positive electrode components under oxidative conditions [45]. While these observations point toward interfacial and mechanical instabilities in the less‐optimized electrolytes, further systematic studies are required to elucidate the precise failure mechanisms and identify the critical factors limiting the high‐rate durability of RMBs. Besides, full‐cell testing with MCE‐MA was also conducted at a higher current density of 200 mA g^−^^1^ (Figure S13). The cell delivers a limited capacity (<50 mAh g^−^^1^), which is primarily attributed to the intrinsically sluggish Mg^2+^ diffusion kinetics in the FeVO cathode, indicating further needs for cathode development with higher rate capability.

Although a comparison with literature employing WCA‐based electrolyte and oxide cathodes could be important to further signify the role of MCE‐MA, direct quantitative comparison of electrochemical performance across different studies remains challenging, as reported values are highly sensitive to variations in cell configuration, electrode preparation, electrolyte composition, and testing protocols. Recent studies on the use of WCA‐based electrolytes for high‐voltage RMBs have shown that achieving stable interphase formation at high voltages remains challenging. In many cases, long‐term cycling is limited by continuous electrolyte decomposition and interfacial instability [3, 5, 46]. In this context, the introduction of MCE‐MA to regulate cation–anion interactions and promote the formation of robust, fluorinated interphases provides insight into improved cycling stability in high‐voltage RMBs systems employing oxide‐based cathode.

In addition to oxide‐based cathodes, the applicability of MCE‐MA was further examined using a benchmark Chevrel‐phase cathode (Mo_6_S_8_) and an organic cathode material, 3,4,9,10‐perylenetetracarboxylic dianhydride (PTCDA). When paired with the Mo_6_S_8_ cathode, the cycling performance of cells employing the base electrolyte and MCE‐MA is largely comparable (Figure S9), with similar discharge–charge overpotentials observed over the initial 20 cycles. This behavior is attributed to the relatively low operating voltage of Mo_6_S_8_, under which oxidative electrolyte degradation is minimal, thereby limiting the advantages conferred by MCE‐MA. Nevertheless, considering the pronounced differences in Mg plating/stripping stability between the base electrolyte and MCE‐MA observed in symmetric cells, the long‐term cycling behavior of low‐voltage cathode systems requires further investigation. For the organic cathode system, cells employing MCE‐MA exhibit improved cycling stability compared to the base electrolyte at a current density of 20 mA g^−1^ (Figure S10). Although the PTCDA‐based full cell displays more rapid capacity fading than the oxide‐based cathode systems, these results demonstrate the broader compatibility of MCE‐MA and highlight organic cathodes as a promising platform for further optimization and mechanistic study.

### 2.5 Interfacial Chemistry After Full‐Cell Cycling

**FIGURE 5:**
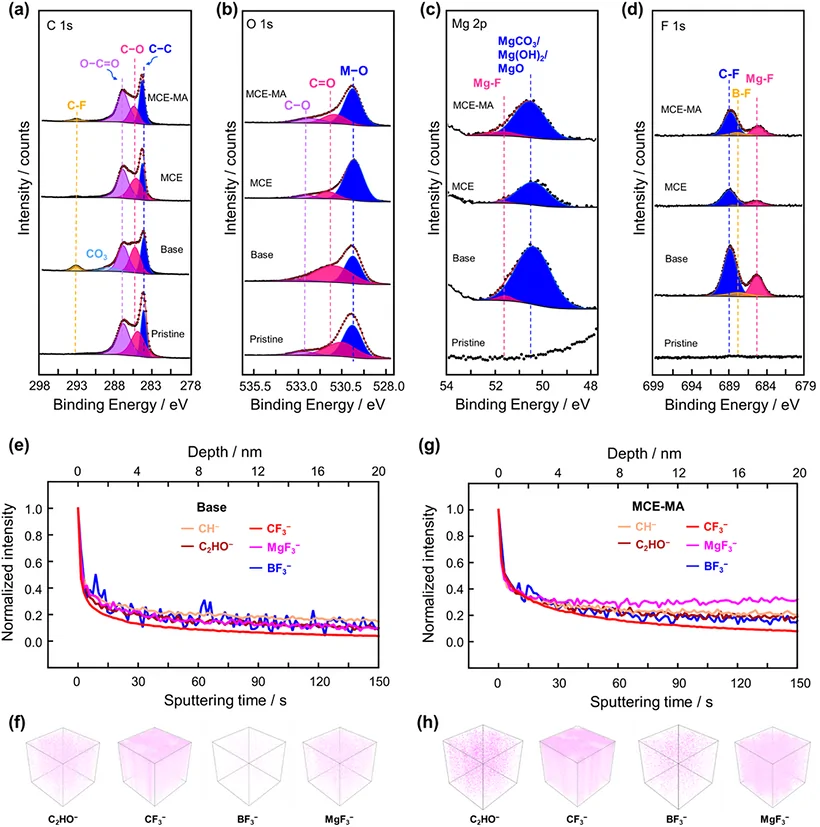
(a – d) Ex situ XPS survey spectra of FeVO cathode after cycling in base electrolyte, MCE, and MCE‐MA; (a) C 1s, (b) O 1s (c) Mg 2p (d) F 1s. (e–h) ToF‐SIMS spectra of FeVO cathode cycled in MCE‐MA compared to base electrolyte; (e) Depth profiling, and (f) 3D mapping of selected ion spectroscopy for base electrolyte. (g) Depth profiling and (h) 3D mapping of selected ion spectroscopy for MCE‐MA.

To critically assess the influence of MCE and MCE‐MA on cathode–electrolyte interphase (CEI) formation, systematic interfacial characterizations were conducted. The interfacial characterizations were performed on electrodes harvested after the same electrochemical protocol, namely after 3 cycles of full‐cell operation at a current density of 10 mA g^−^^1^, rather than after prolonged cycling. This condition was intentionally selected to ensure comparable interphase formation while avoiding significant degradation or thickness accumulation associated with long‐term cycling. Figure 5a–d shows the XPS spectra of FeVO electrodes in the pristine state and after cycling in the base, MCE, and MCE‐MA electrolytes. In the C 1s spectra, a carbonate‐related peak (∼290 eV) appears after cycling in the base electrolyte (Figure 5a) but is absent in MCE and MCE‐MA, indicating that the mixed‐anion systems effectively suppress solvent‐derived decomposition. All electrolytes exhibit C–F features with varying intensities, suggesting localized and nonuniform interphase formation.

The O 1s spectra reveal broadly similar CEI compositions among the three systems, except for a more pronounced C─O signal in the base electrolyte (Figure 5b), consistent with greater organic accumulation observed in the C 1s spectra. The Mg 2p spectra confirm the presence of Mg(CO_3_)_2_, Mg(OH)_2_, and/or MgO species across all samples (Figure 5c), accompanied by minor Mg─F components. In the F 1s region (Figure 5d), the base electrolyte exhibits relatively stronger C─F, Mg─F, and B─F peaks than MCE and MCE‐MA, reflecting a more extensive anion‐derived fluorinated interphase. These results contrast with the SEI analysis, where MCE and MCE‐MA displayed higher fluorinated species intensity, correlating with their enhanced Mg plating/stripping stability. However, XPS alone provides limited insight into CEI robustness, as fluorinated species buried beneath several nanometers of surface layers may not be fully detected due to signal attenuation of F 1s photoelectrons.

To verify this, detailed depth‐profiling analyses using ToF‐SIMS were performed on FeVO electrodes cycled in MCE‐MA, alongside comparative measurements with the base electrolyte (Figure 5e–h). The ToF‐SIMS depth profiles (Figure 5e,f) reveal that fluorine‐containing species (MgF_3_^−^, CF_3_^−^, and BF_3_^−^) persist much deeper within the CEI of MCE‐MA, whereas their intensity in the base electrolyte rapidly diminishes with sputtering time. The abundance of organic fragments remains comparable between the two systems, suggesting that the fluorinated interphase in MCE‐MA is not only chemically distinct but also physically thicker and more uniform. In contrast, the fluorinated components in the base electrolyte are concentrated near the surface, forming a thinner and patchier interphase. 3D reconstructions (Figure 5g,h) further confirm that Mg–F and B–F species form a more continuous, extended, and spatially homogeneous interphase layer in MCE‐MA, underscoring its superior interfacial robustness.

**FIGURE 6:**
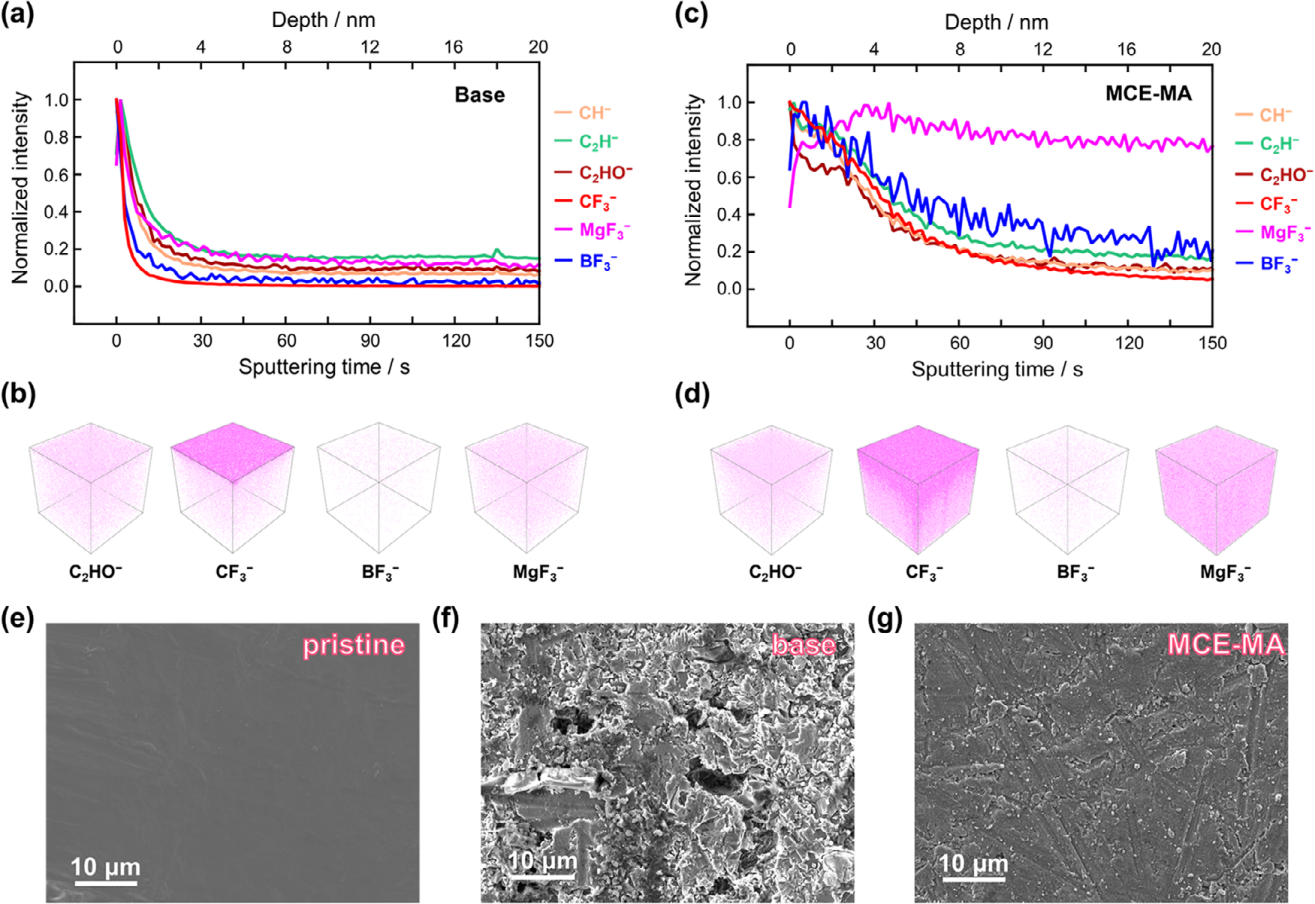
(a–d) ToF‐SIMS spectra and 3D mapping of selected ion spectroscopy of Mg metal anode cycled RMBs full cell with different electrolytes; (a,b) base electrolyte and (c,d) MCE‐MA. (e–g) SEM images of Mg metal anode at (e) pristine stage, and after cycled in RMBs full cell with different electrolytes; (f) base electrolyte and (g) MCE‐MA.

Interfacial processes at the cathode are expected to influence not only CEI stability but also the evolution of the anode interphase and the homogeneity of Mg plating/stripping during full‐cell operation [3]. To probe this coupling, ToF‐SIMS was further employed to examine the Mg anode interphase after full‐cell cycling in MCE‐MA, with direct comparison to the base electrolyte. In the MCE‐MA system, fluorinated species are detected not only at the immediate Mg surface but also throughout deeper regions of the interphase (Figure 6c), indicating the formation of a more integrated and chemically continuous interfacial layer. By contrast, the base electrolyte exhibits a more surface‐confined and spatially heterogeneous distribution of fluorinated species (Figure 6a). 3D ToF‐SIMS reconstructions further emphasize this distinction: MCE‐MA yields a continuous and uniformly distributed interphase across the Mg surface (Figure 6d), whereas the base electrolyte produces a thin and uneven interphase (Figure 6b). These chemical differences are corroborated by morphological observations. SEM images of Mg metal in the pristine state and after full‐cell cycling are shown in Figure 6e–g. Following cycling in the base electrolyte, the Mg surface becomes markedly rough, consistent with localized and inhomogeneous Mg plating/stripping, similar to what has been observed in the symmetric Mg|Mg cell test (Figure 6f). In contrast, Mg metal cycled in MCE‐MA retains a comparatively smooth and compact surface morphology (Figure 6g), indicative of more uniform Mg deposition and dissolution.

### 2.6 Toward Broader Electrolyte Component

To provide additional insight into future electrolyte engineering strategies based on mixed‐coordination chemistries, we briefly examined the effects of introducing a different ether‐based molecular additives such as 1,1,2,2‐tetrafluoroethyl 2,2,3,3‐tetrafluoropropyl ether (TTE), ethyl 1,1,2,2‐tetrafluoroethyl ether (ETFE), 1‐fluoro‐2‐(2‐fluoroethoxy)ethane (HFE), and 2,2‐bis(trifluoromethyl)‐1,3‐dioxolane (BisTMD).

Symmetric Mg|Mg cell tests revealed that replacing the molecular additive of BisTFE with TTE maintained stable Mg plating/stripping for more than 500 cycles (Figure S11), while other additives performed differently. However, when evaluated in full cells using the FeVO cathode, the electrolyte containing TTE experienced rapid degradation and undesirable reactions at moderate operating voltages (Figure S12). The underlying mechanisms responsible for this instability remain uncertain and require further systematic investigation. Above all, these findings highlight the critical need to balance Mg plating/stripping performance with the stability of high‐voltage full cells when incorporating broader electrolyte components.

## 3 Conclusion

In this work, we establish a mixed‐coordination electrolyte integrated with a molecular additive (MCE‐MA) as a new electrolyte design concept for stabilizing high‐voltage rechargeable magnesium batteries. Rather than relying on electrolytes with a single salt, this strategy deliberately combines weakly coordinating salts with distinct cation–anion coordination characteristics and a functional molecular additive to simultaneously regulate Mg^2+^ solvation and interphase formation. Specifically, the coexistence of Mg[B(hfip)_4_]_2_ and a trace amount of Mg[B(tfe)_4_]_2_ enables controlled anion participation in the solvation structure, while the BisTFE additive promotes the formation of a homogeneous and robust interphase.

As a result of this cooperative electrolyte design, MCE‐MA delivers markedly improved Mg plating/stripping stability, sustaining over 250 h of operation in Mg|Mg symmetric cells, whereas the base electrolyte (0.3 m Mg[B(hfip)_4_]_2_ in G2) short‐circuits within 100 h. Moreover, MCE‐MA enables long‐term cycling of RMBs full cell with oxide‐based cathodes for 200 cycles, in sharp contrast to the rapid failure of the base electrolyte within 30 cycles. Mechanistic analyses reveal that the MCE–MA provides a practical route to forming thicker and more robust fluorinated CEI and SEI layers compared to the base electrolyte, thereby improving the homogeneity of Mg plating and stripping during full‐cell operation.

Beyond the specific electrolyte formulation studied here, this work demonstrates that integrating mixed‐coordination chemistry with molecular additive engineering constitutes a generalizable strategy for electrolyte design. This approach establishes a new framework for decoupling ion transport from interphase stability and offers broad guidance for the development of durable, high‐voltage rechargeable magnesium batteries and other multivalent energy‐storage systems.

## 4 Experimental Section

### 4.1 Materials

Tetrahydrofuran (THF), Dimethoxyethane (ethylene glycol dimethyl ether, DME), and diglyme (diethylene glycol dimethyl ether, G2) were purchased from Kanto Chemical. Trifluoroethanol (tfe, ≥99.0%) was purchased from Nacalai Tesque, Inc. All of the above solvents were treated with molecular sieves for a minimum of one day prior to use, and their water content was less than 10 ppm as measured by Karl‐Fischer moisture titration (MKC710, KEM). 1 m Zn(BH_4_)_2_ in THF was purchased from BetaPharma (Shanghai) Co., Ltd. and directly used without further treatment. For the electrolyte preparation, bis(2,2,2‐trifluoroethyl) ether (BisTFE) was purchased from Sigma–Aldrich (98%), and 1,1,2,2‐tetrafluoroethyl 2,2,3,3‐tetrafluoropropylether (TTE) was purchased from TCI Chemicals (>95.0%). Both solvents were used as received. For cathode preparation, NH_4_VO_3_ (≥ 99.0%) and Fe(NO_3_)_3_.9H_2_O (≥ 98%) were purchased from Sigma–Aldrich. PTCDA (> 98%) was purchased from TCI Chemicals and used as received. Cu_2_Mo_6_S_8_, a precursor of Mo_6_S_8_ cathode material, was purchased from Kojundo Chemical Laboratory Co., Ltd.

### 4.2 Salt Synthesis

Mg[B(hfip)_4_]_2_ was synthesized by transmetalation reaction using di‐*n*‐butyl magnesium following the previously reported method [8].

Mg[B(tfe)_4_]_2_ was synthesized via dehydrogenation of Mg(BH_4_)_2_. Specifically, Mg(BH_4_)_2_ in THF solution was prepared first by precipitation reaction from 1 m Zn(BH_4_)_2_ in THF (BetaPharma (Shanghai) Co., Ltd.). In a reaction flask, 50 mL of 1 m Zn(BH_4_)_2_ in THF was mixed with an additional 50 mL of THF. Then, 1.2 g of Mg turnings were gradually added to the solution and stirred at room temperature for one week. During this period, Zn‐based precipitates gradually formed. The resulting Mg(BH_4_)_2_/THF solution was obtained by filtering the mixture. Next, 20 mL of the filtered Mg(BH_4_)_2_/THF solution was transferred into a reaction flask containing 20 mL of DME. 7.2 mL of trifluoroethanol (tfe, 99%, Nacalai Tesque, Inc.) was added slowly to the mixture, in which hydrogen evolution could be observed, followed by stirring for 20 h. Finally, the solvent was removed by vacuum drying at 45°C for at least 12 h. The salt was collected and stored in an Ar‐filled glovebox, while the purity was confirmed by ^1^H NMR (JNM‐ECA 400, JEOL, ^1^H resonance frequency = 400 MHz). The crystallographic structures of Mg[B(hfip)_4_]_2_ and Mg[B(tfe)_4_]_2_ were reproduced from CIF files deposited in the Cambridge Crystallographic Data Centre (CCDC 1537493 for Mg[B(hfip)_4_]_2_ and CCDC 2091598 for Mg[B(tfe)_4_]_2_).

### 4.3 Electrolyte Preparation

The base electrolyte, 0.3 m Mg[B(hfip)_4_]_2_ in G2, was prepared by dissolving the Mg[B(hfip)_4_]_2_ salt in G2 solvent with 1 h stirring at room temperature in an Ar‐filled glovebox. G2 (>99.5 %, Kanto Chemical CO., INC., Japan) solvent was treated with molecular sieves overnight prior to electrolyte preparation. MCE and MCE‐MA were prepared following the same method according to the composition in Table S1. The water content of as‐prepared electrolytes was in the range of 30 – 150 ppm, as measured by Karl–Fischer moisture titration (MKC710, KEM). The Raman spectra of as‐prepared electrolytes were measured by a Raman spectrometer (NRS‐4500, Jasco).

### 4.4 Cathode Preparation

FeV_3_O_9_·1.1H_2_O (FeVO) powder was prepared following the previous method using NH_4_VO_3_ and Fe(NO_3_)_3_.9H_2_O as precursor [43]. Specifically, 24 mmol of NH_4_VO_3_ was stirred in 800 mL of distilled water at 90°C for 1 h until it dissolved completely, namely solution A. Separately, 8 mmol of Fe(NO_3_)_3_∙9H_2_O was dissolved in 80 mL of distilled water, namely solution B. Solution B was added slowly into solution A without stirring, producing yellow‐colored insoluble colloidal products. Then, the mixture was aged at 90°C for 24 h. The resulting brown precipitates were filtered, then thoroughly washed with distilled water multiple times and dried at 80°C for 24 h under vacuum.

Mo_6_S_8_ powder was produced by Cu leaching from Cu_2_Mo_6_S_8_ following the reported procedure and stored under dry air [47].

Cathode material was prepared by mixing active material, Acetylene Black, and PVDF binder with a mass ratio of 8: 1: 1, dispersed in *N*‐methyl‐2‐pyrrolidone (NMP), and cast onto carbon‐coated aluminum foil as a current collector. For the XPS analysis, polyacrylonitrile (PAN) binder was used instead of PVDF to avoid F 1s overlapping. The loading mass of the cathode was 0.8 – 1.5 mg/cm^2^.

### 4.5 Electrochemical Characterization

A two‐electrode type cell (SB2A‐EC‐frontier) was used in symmetric cell, asymmetric cell, and full cell tests [48, 49, 50]. For the symmetric and asymmetric cell test, a glass fiber separator (GF/D, Whatman) (2.01 cm^2^) was used, while for the Mg deposition test, a polyethylene (PE, ◎wscope, SB‐20D) separator was used. For the full cell, cathode active material (2.01 cm^2^), a glass fiber separator (GF/D, Whatman) (2.01 cm^2^), and polished Mg metal (2.01 cm^2^) were assembled in a two‐electrode cell for full cell test. The amount of electrolyte for each cell was 250 µL. The LSV test was performed using a home‐made beaker‐type cell. Ag/Ag^+^ was used as a reference electrode and calibrated as 2.49 V vs. Mg/Mg^2+^ [9].

For XRD sampling of the Mg deposit, graphite‐coated aluminum foil is used. The deposition was done with a current density of 0.5 mA cm^−2^ for 2 h. The resulting deposit was washed with THF and dried inside an Ar‐filled glovebox.

The full cell discharge‐charge measurement was conducted using a battery cycler (HJ1001SD8 C, HD Meiden Hokuto, Japan). While EIS was conducted using EC‐Lab software on a Biologic VMP3 multichannel potentiostat (Biologic Science Instruments SAS). The cut‐off voltage of the full cell was 0.8–3.3 V, with 3 h constant‐current constant voltage mode (CCCV) during charge for 10 mA g^−1^, and 1 h for 100 mA g^−1^.

### 4.6 Cathode and Anode Characterization

The surfaces of the Mg anode and FeVO cathode after electrochemical cycling were characterized by X‐ray photoelectron spectroscopy (XPS, VersaProbe II, ULVAC‐PHI), time‐of‐flight secondary ion mass spectrometry (ToF‐SIMS, ToF‐SIMS5‐AD‐GCIB), and scanning electron microscopy (SEM, SU8200, Hitachi, Japan) equipped with energy‐dispersive X‐ray spectroscopy (EDX). ToF‐SIMS measurements were performed using a 30 kV Bi_3_^+^ primary ion beam, with a sputtering rate calibrated to 4 nm min^−1^ based on a SiO_2_ reference film. All electrochemical cells were disassembled inside an Ar‐filled glovebox. The retrieved electrodes were rinsed three times with tetrahydrofuran (THF) and dried inside the glovebox for several hours prior to analysis. All interfacial characterizations, including sample transfer, were carried out under strictly air‐ and moisture‐free conditions.

## Funding

This work was financially supported by the GteX Program Japan (Grant Number JPMJGX23S1) of the Japan Science and Technology Agency.

## Conflicts of Interest

The authors declare no conflicts of interest.

## Supporting information




**Supporting File**: advs75749‐sup‐0001‐SuppMat.docx.

## Data Availability

Research data are not shared.
