# Supplementary material for: Mixed‐Coordination Electrolytes With Molecular Additives for Robust Interphases in High‐Voltage Rechargeable Magnesium Batteries
Source: Adv Sci (Weinh). 2026 May 25:e75749. Online ahead of print. doi: 10.1002/advs.75749 (PMC13336113; doi:10.1002/advs.75749)
Supplement: Supplementary file 1 — Supporting File: advs75749‐sup‐0001‐SuppMat.docx. [file ADVS-9999-e75749-s001.docx]

**Supporting Information**

Mixed-Coordination Electrolytes with Molecular Additives for Robust Interphases in High-Voltage Rechargeable Magnesium Batteries

Dedy Setiawan, ^a^ and Toshihiko Mandai*^, a^

^a^ Functional Electrolyte Synthesis Team, Research Center for Energy and Environmental Materials (GREEN), National Institute for Materials Science (NIMS), Tsukuba, Ibaraki 305-0044, Japan

*[Setiawan.Dedy@nims.go.jp](mailto:Setiawan.Dedy@nims.go.jp), [Dedysetiawan.indonesia@gmail.com](mailto:dedysetiawan.indonesia@gmail.com), [Mandai.Toshihiko@nims.go.jp](mailto:Mandai.Toshihiko@nims.go.jp)

**
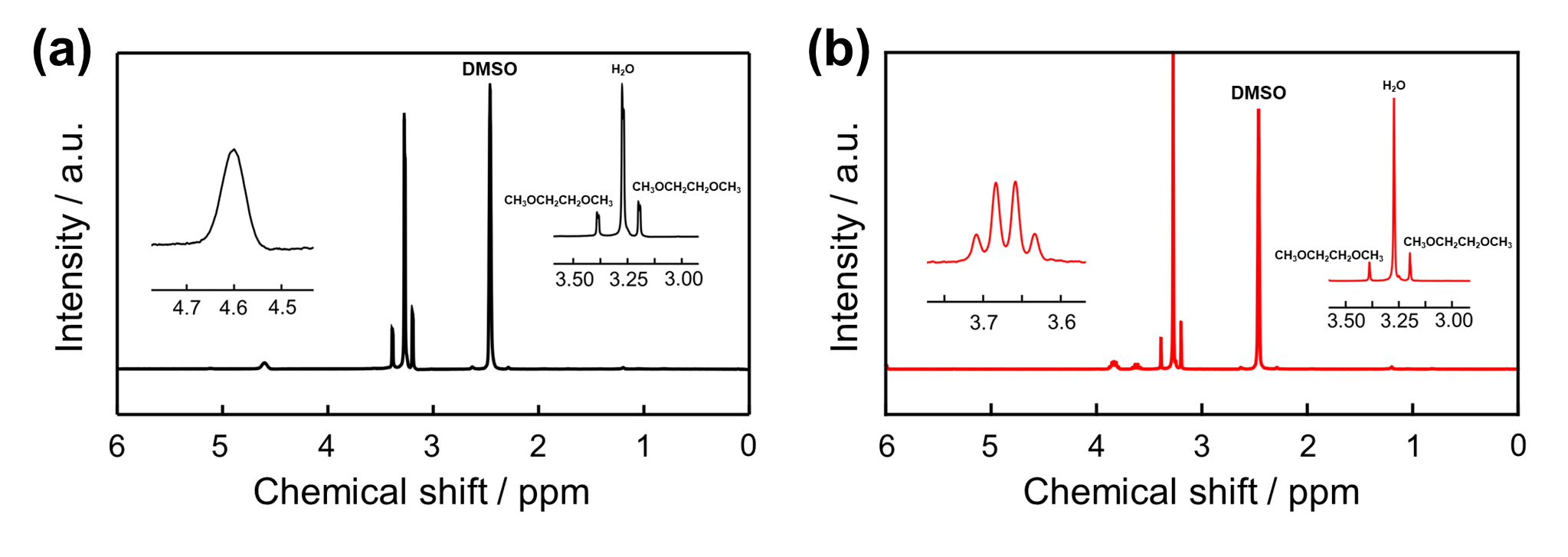
**

**Figure S1.** ^1^H NMR spectra of as-prepared salts (a) Mg[B(hfip)_4_]_2_ (b) Mg[B(tfe)_4_]_2_.

**Table S1.** Electrolyte formulation

| Electrolyte | Salt Concentration (M) | | **Solvent Volume Ratio (%)** | |
| --- | --- | --- | --- | --- |
|  | Mg[B(hfip)_4_]_2_ | Mg[B(tfe)_4_]_2_ | **G2** | **BisTFE** |
| Base electrolyte | 0.3 | 0 | **100** | **0** |
| MCE | 0.3 | 0.005 | **100** | **0** |
| MCE-MA | 0.3 | 0.005 | **99** | **1** |


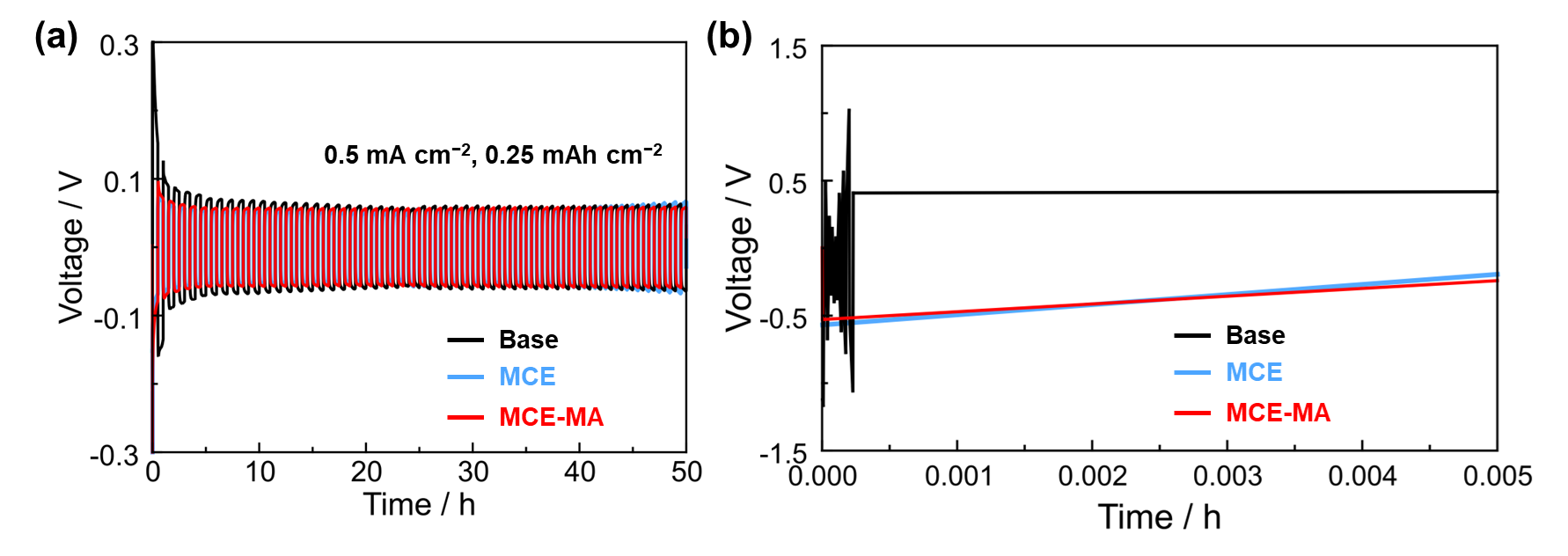


**Figure S2.** (a) Discharge/charge profile of symmetric cell Mg|Mg cells cycled for 50 hours with base electrolyte, MCE, and MCE-MA for solid electrolyte interphase (SEI) analysis. (b) Early stage of discharge/charge profile of symmetric cell Mg|Mg cells with base electrolyte, MCE, and MCE-MA; The cell with base electrolyte undergoes activation process before plating/stripping process proceed. The figure was drawn with wider voltage range for clarity.


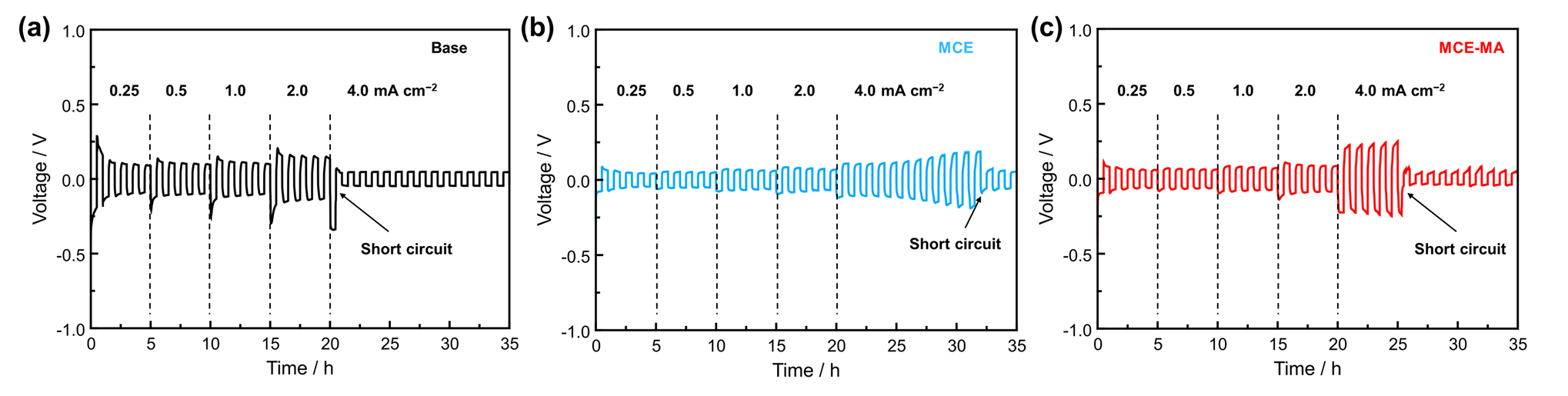


**Figure S3.** Rate performance of symmetric Mg|Mg cell with (a) base electrolyte, (b) MCE, and (c) MCE-MA. The discharge/charge period is 30 min (1 hour per cycle).


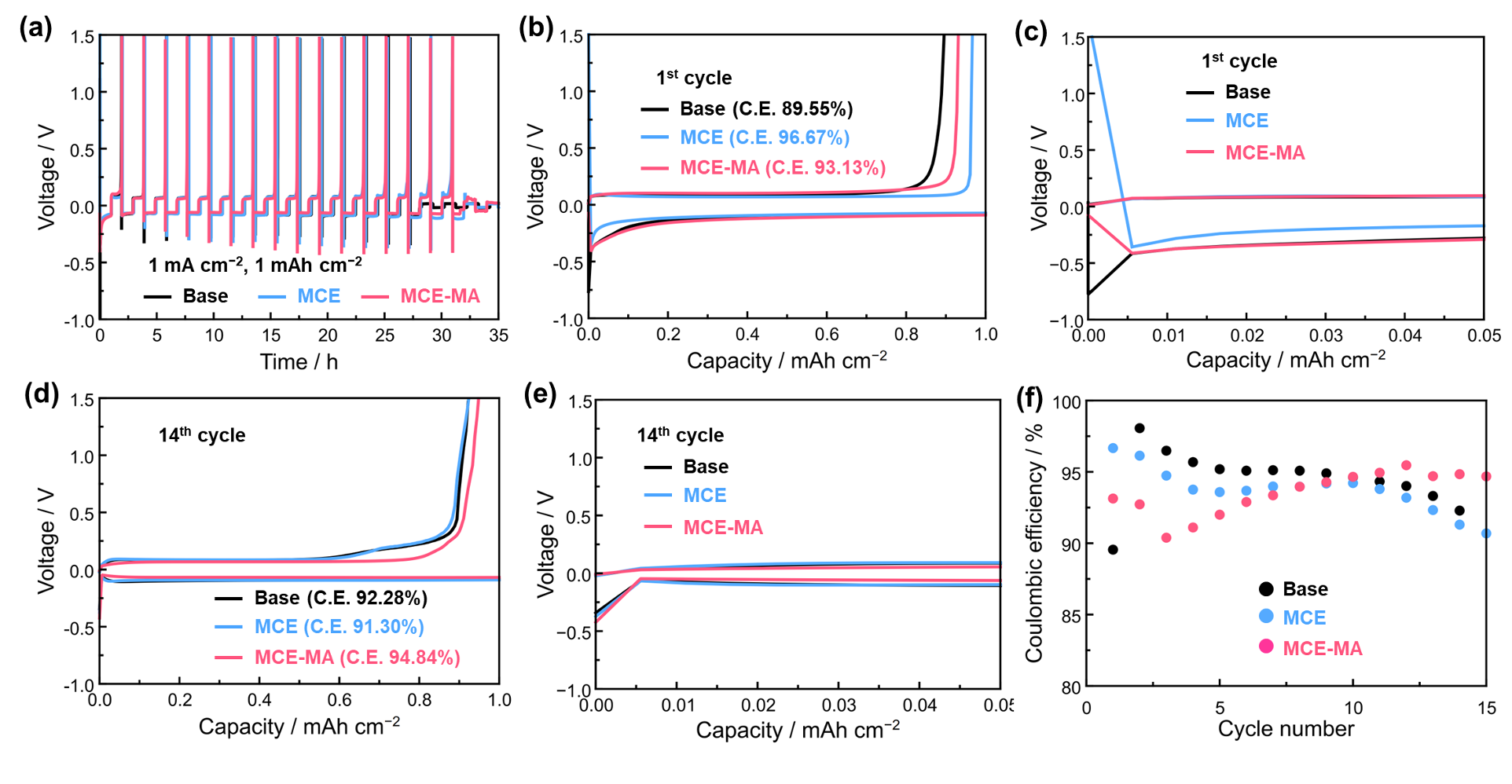


**Figure S4.** (a) Galvanostatic discharge/charge profile of asymmetric Cu|Mg cells with base electrolyte, MCE, and MCE-MA cycled at 1 mA cm^−2^_,_ 1.0 mAh cm^−2^. (b - e) Representative cycle of asymmetric Cu|Mg cells with magnified profiles, (b) 1^st^ cycle (c) magnified profile of 1^st^ cycle and (d) 14^th^ cycle (e) magnified profile of 14^th^ cycle. (f) Coulombic efficiency of Mg plating/stripping in asymmetric Cu|Mg cells over cycles.

It is worth noting that although the asymmetric Cu|Mg cells displayed similar lifetimes before short-circuiting across all electrolytes, the symmetric configuration provides a more direct measure of Mg plating/stripping stability. This discrepancy highlights the substrate-dependent nature of Mg electrodeposition. In Cu|Mg cells, Mg nucleation occurs on an inert foreign substrate, leading to non-uniform growth and electrically isolated deposits that obscure electrolyte-dependent effects.^[1]^  In contrast, symmetric Mg|Mg cells eliminate this nucleation barrier, allowing Mg to cycle on its native surface, where the electrolyte–metal interphase dominates performance.^[2]^  Under these conditions, the stabilizing influence of MCE-MA becomes evident, enabling significantly prolonged cycling compared to the other systems. This comparison underscores that asymmetric cells primarily probe nucleation and morphology on foreign substrates, whereas symmetric cells more accurately assess interfacial stability on Mg metal itself. Therefore, both asymmetric and symmetric configurations offer complementary but non-interchangeable insights and relying solely on a single test may underestimate the true interfacial benefits of advanced electrolyte design for metal anodes.^[3]^


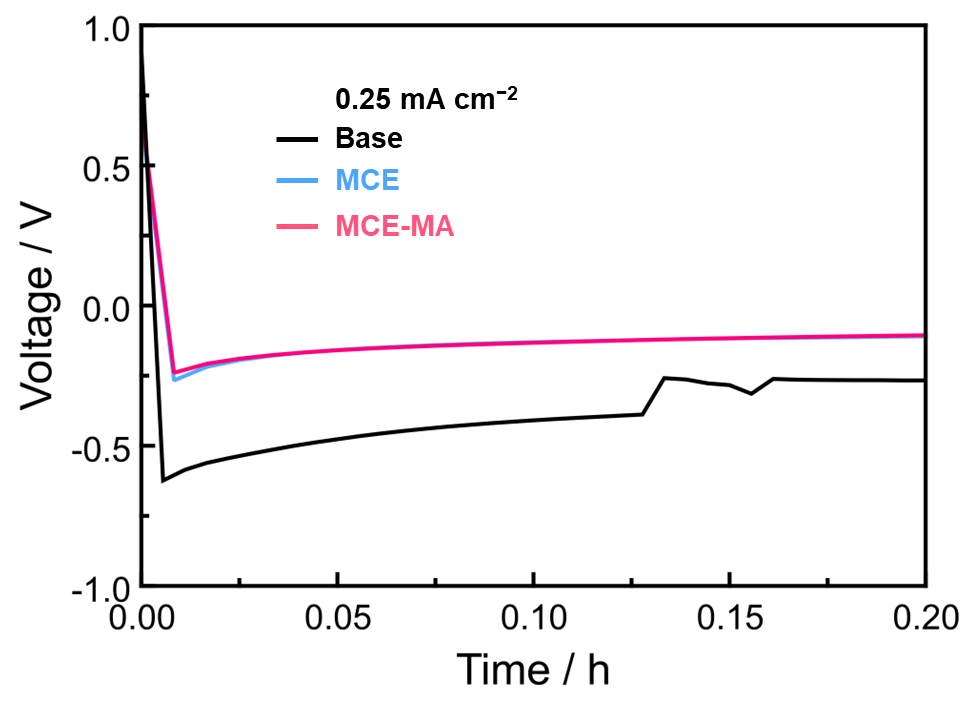


**Figure S5.** Early-stage discharge profile of Mg deposition on Cu working electrode with different electrolytes at 0.25 mA cm^−2^ indicates nucleation overpotential differences


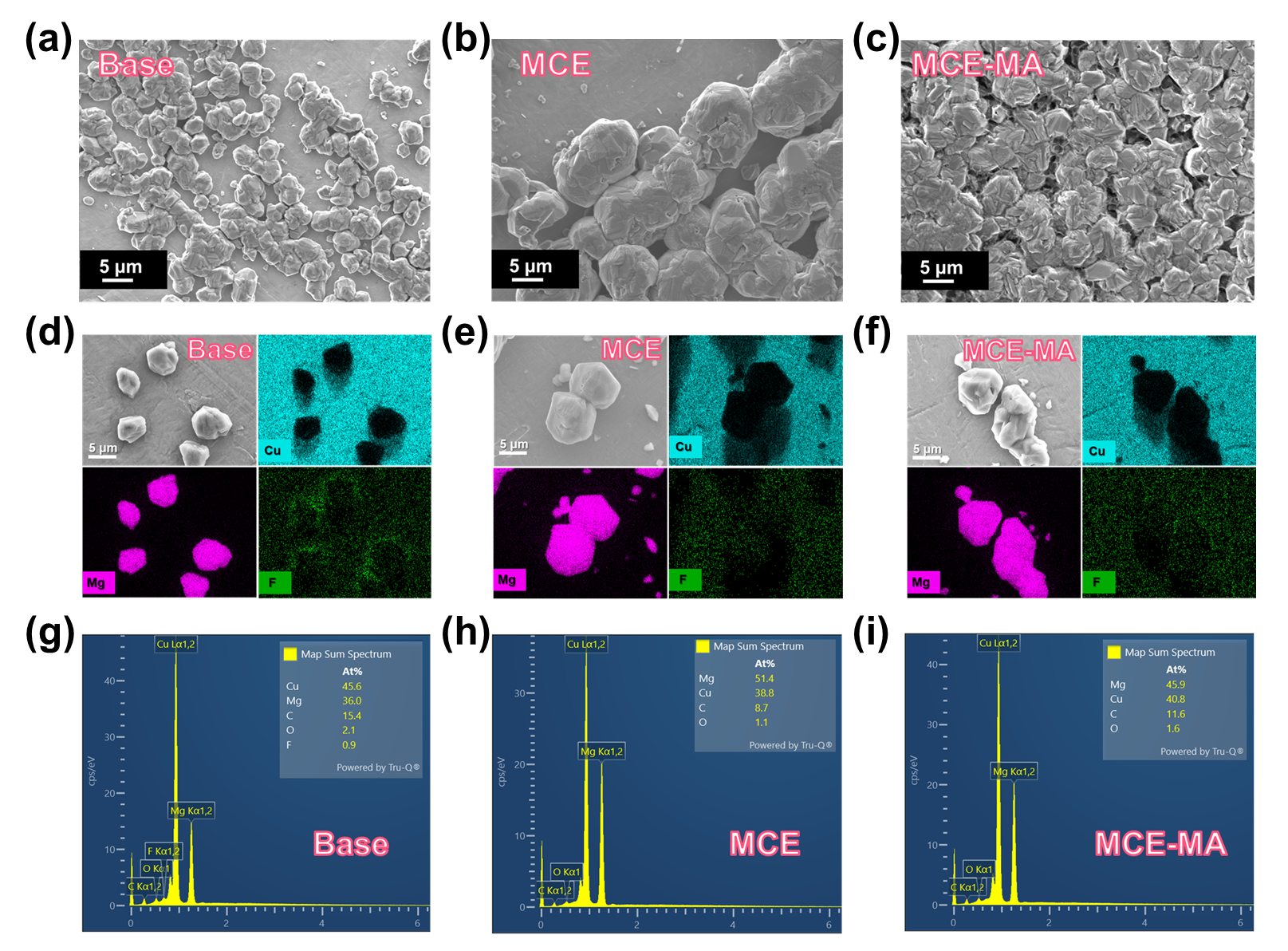


**Figure S6.** (a – c) SEM images of Mg deposits on Cu foil with different electrolytes.


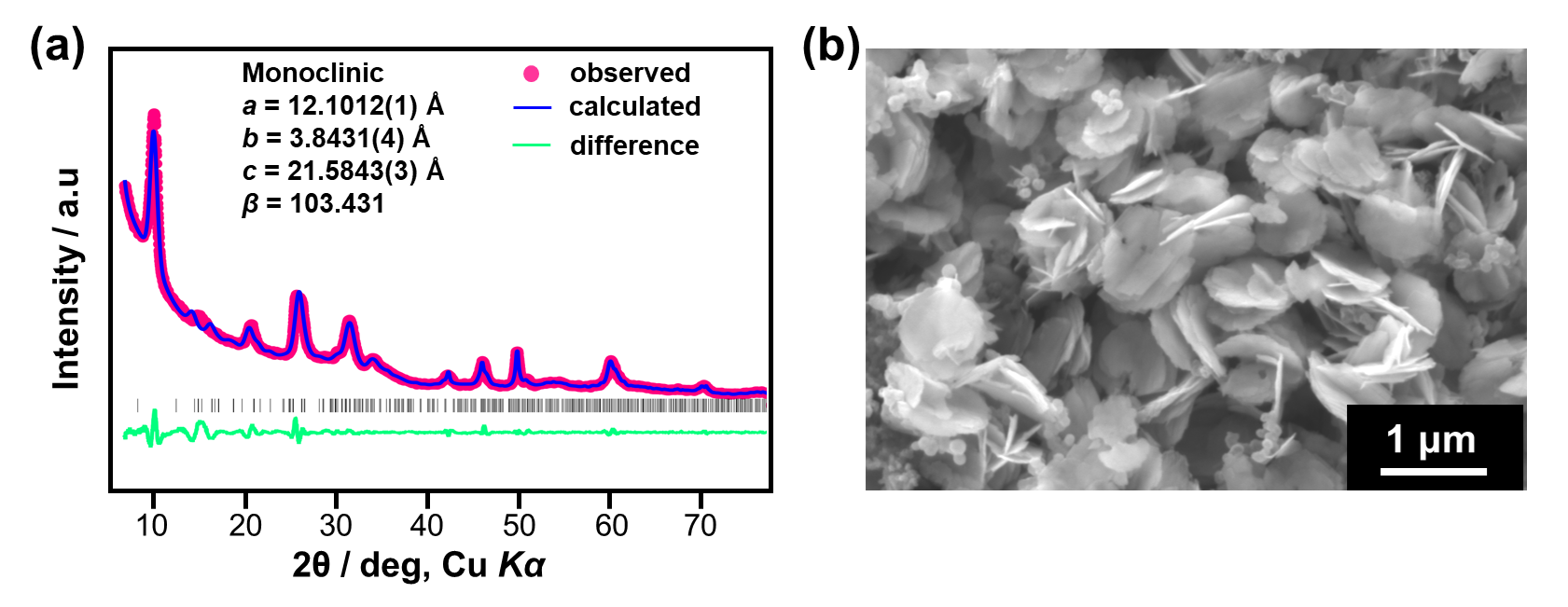


**Figure S7.** Characterization of FeV_3_O_9_•1.1H_2_O (FeVO) cathode; (a) Le Bail fitting of X-ray diffraction data, (b) SEM image.


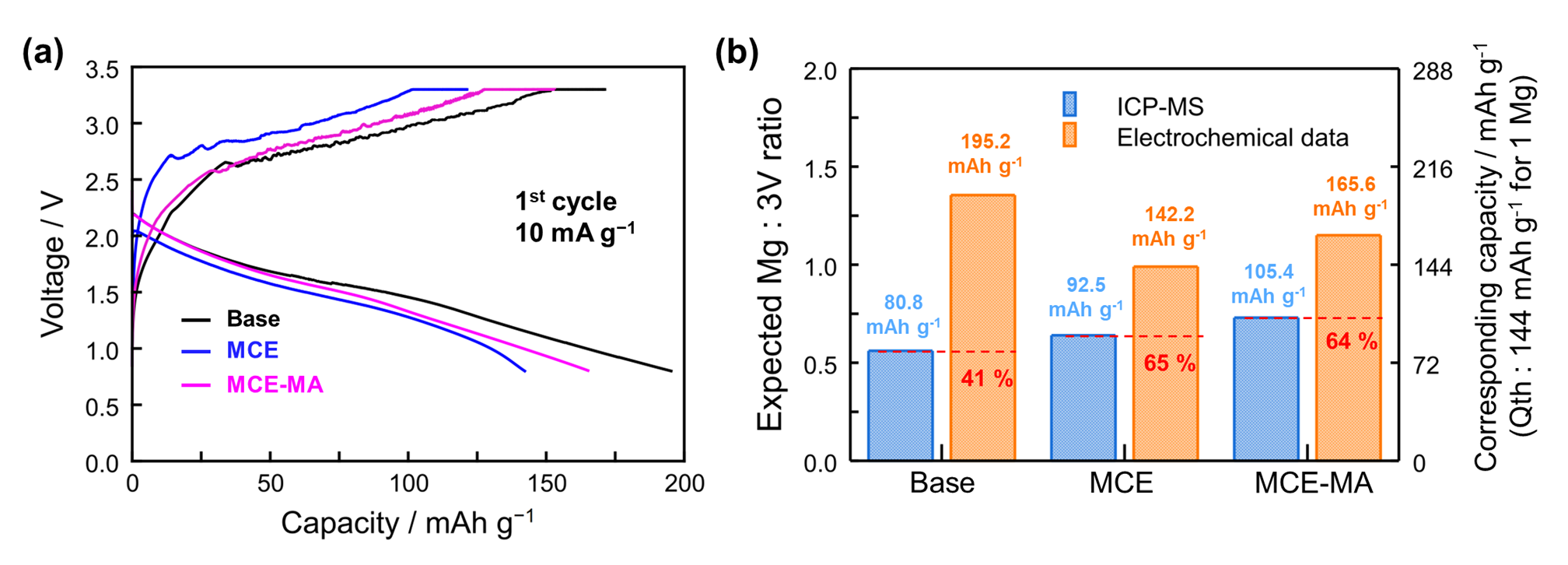


**Figure S8.** (a) Discharge-charge profile comparison of RMBs full cell with FeVO cathode comprising base electrolyte, MCE, and MCE-MA in the 1^st^ cycle at 10 mA g^−1^. (b) Mg : V ratio analysis of discharge electrode at different electrolytes measured by ICP-MS.


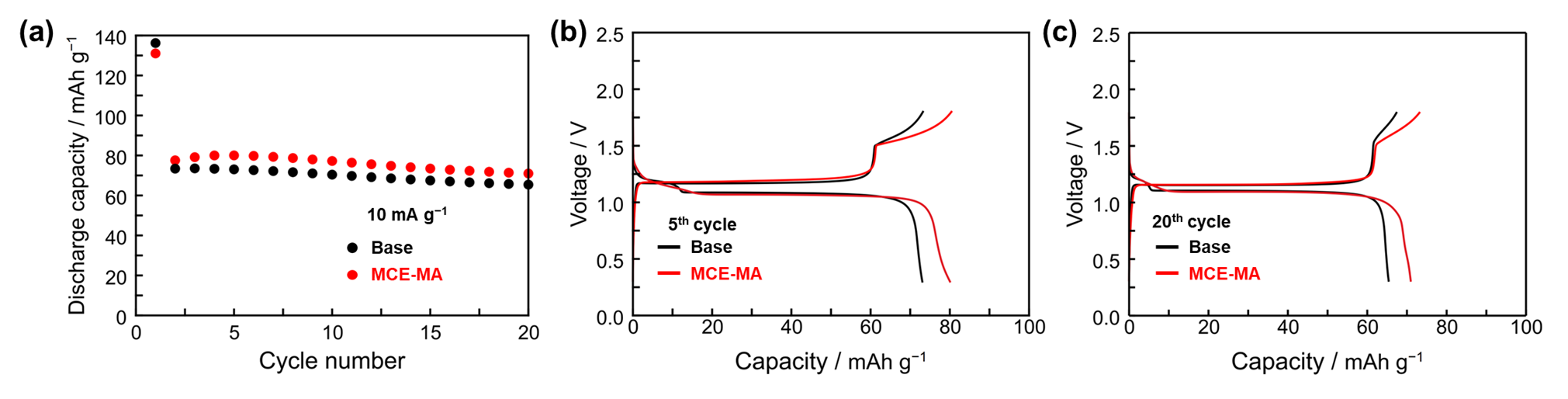


**Figure S9.** (a) Cycle performance comparison of base electrolyte and MCE-MA RMB full cell with Mo_6_S_8_ cathode at 10 mA g^−1^. (b & c) Representative discharge/charge profile; (b) 5^th^ cycle and (c) 20^th^ cycle.


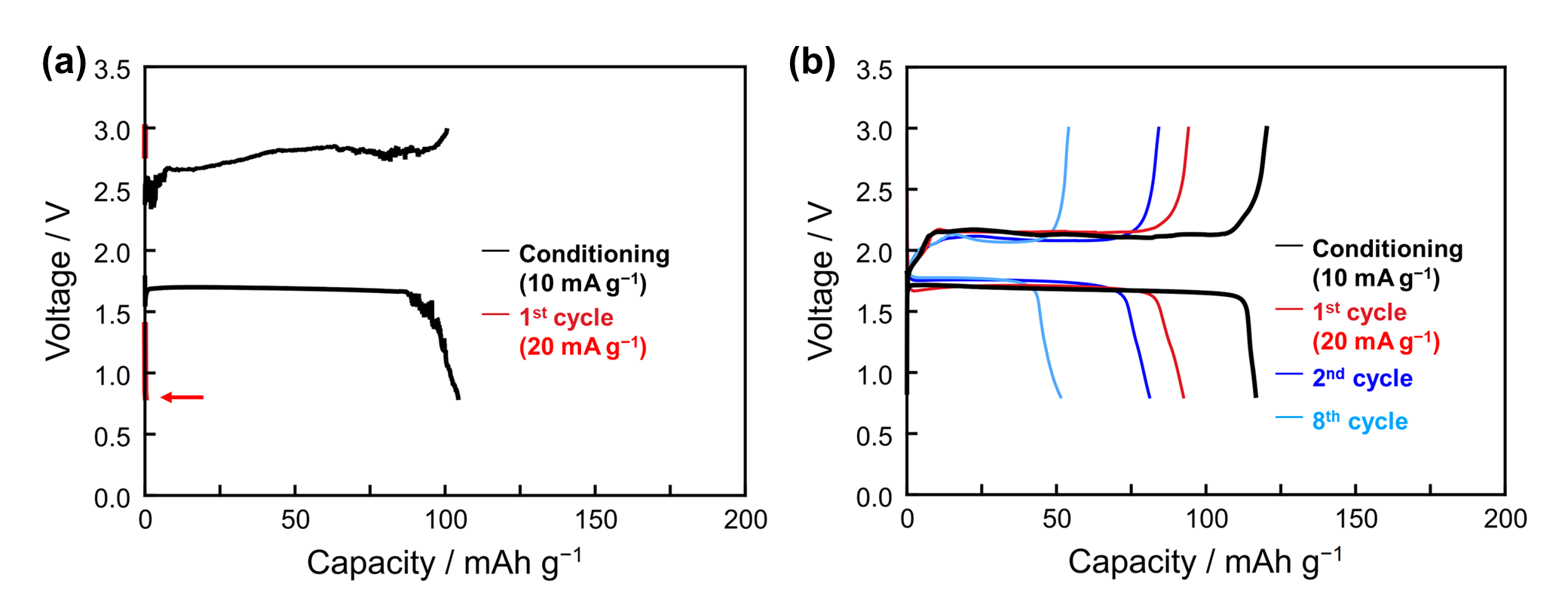


**Figure S10.** Discharge-charge profile comparison of RMBs full cell using PTCDA cathode; (a) with base electrolyte (b) with MCE-MA (1^st^ cycle at 10 mA g^−1^ and the following cycle at 20 mA g^−1^)


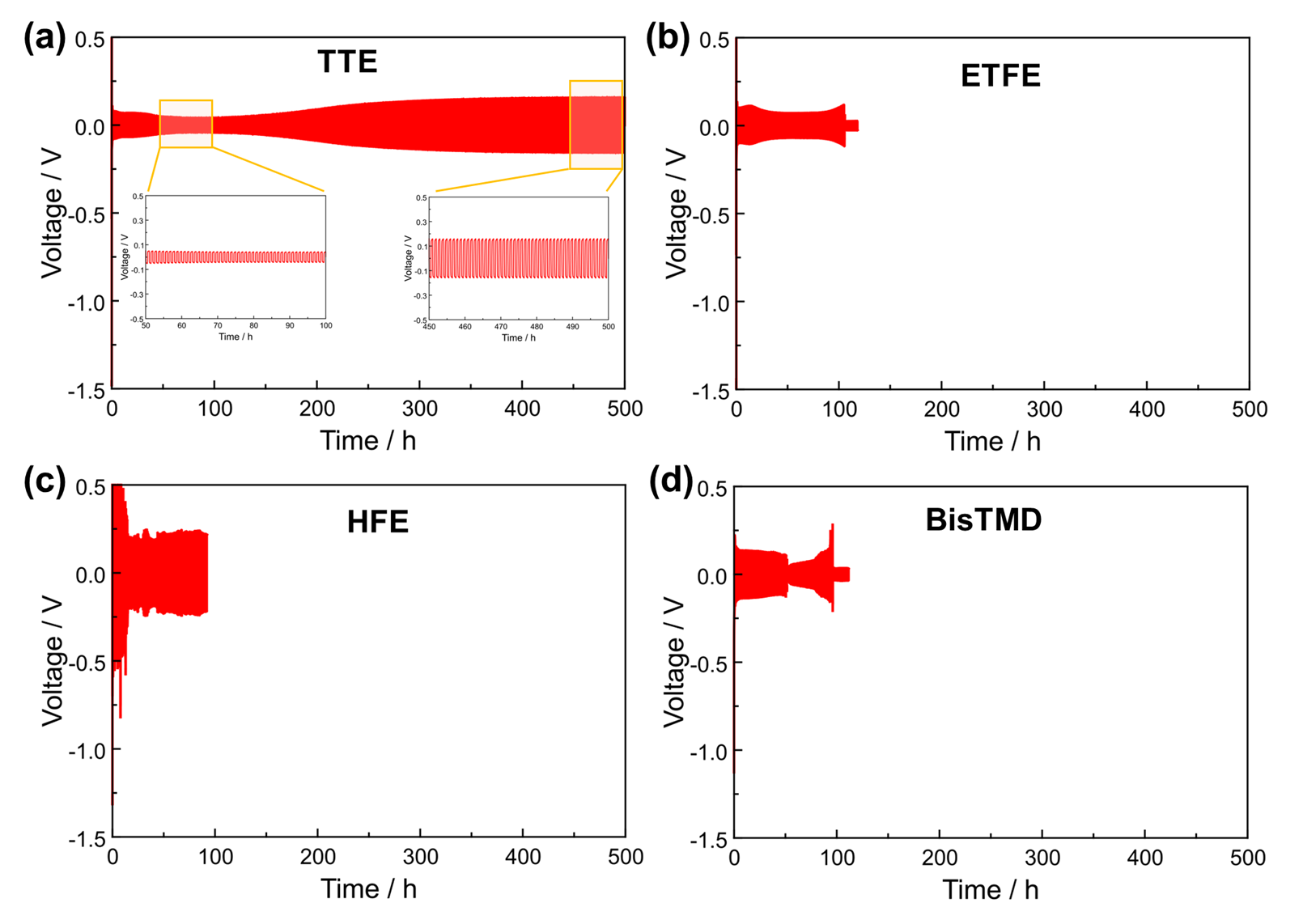


**Figure S11.** Discharge-charge profile symmetric Mg|Mg cell with MCE-MA using different molecular additives instead of BisTFE. (a) TTE, (b) ETFE, (c) HFE, (d) BisTMD.


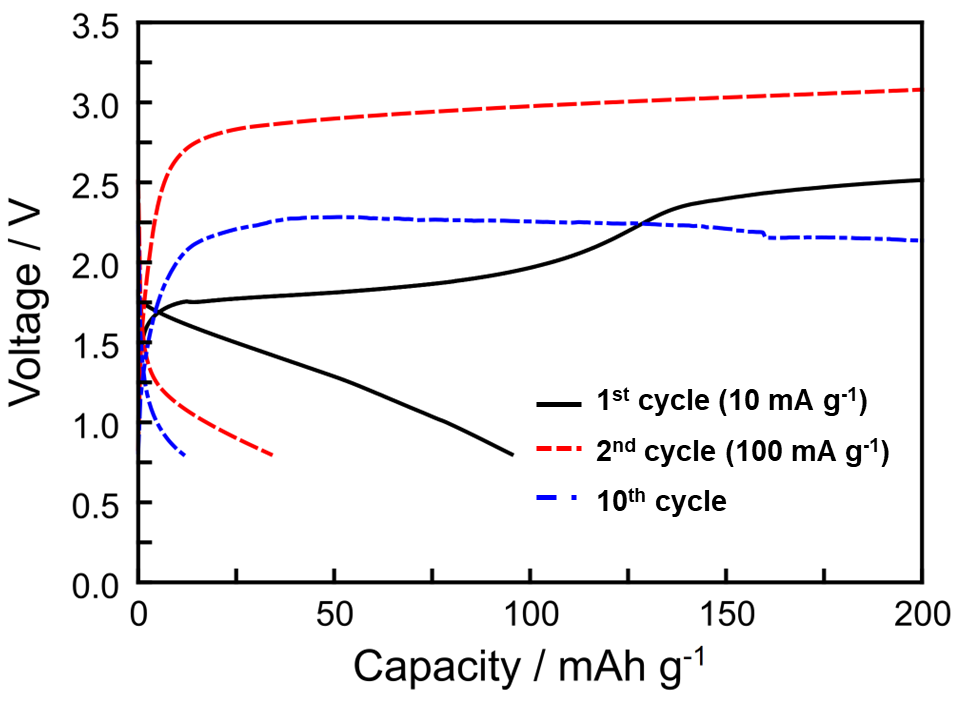


**Figure S12.** Discharge-charge profile of RMBs full cell with FeVO cathode and MCE-MA using TTE molecular additive.


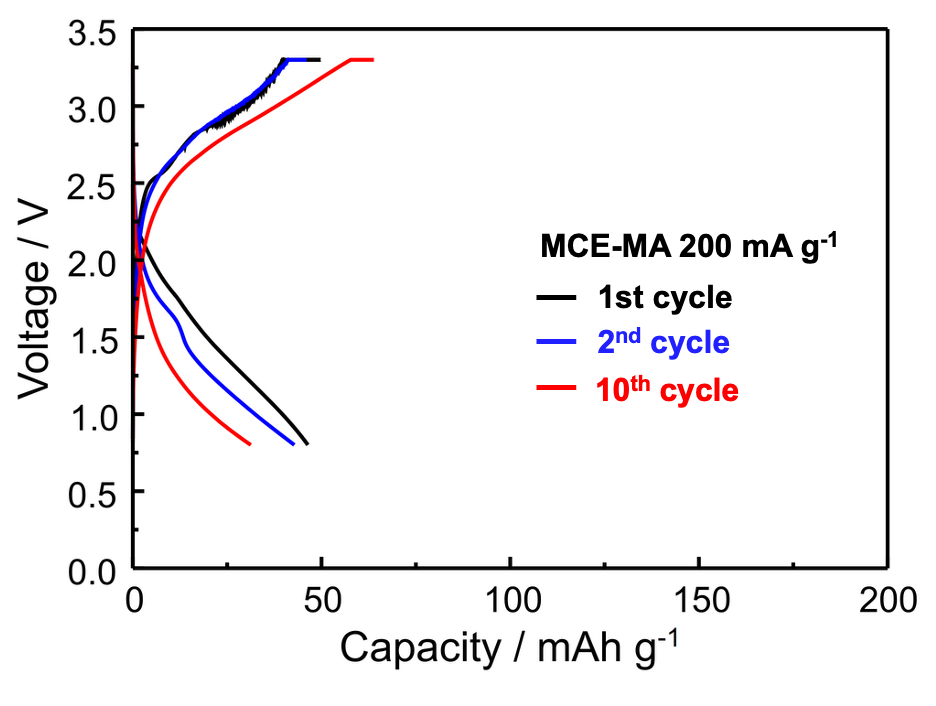


**Figure S13.** Discharge-charge profile of RMBs full cell with FeVO cathode and MCE-MA cycled at 200 mA g^−1^, after conditioning at 10 mA g^−1^.


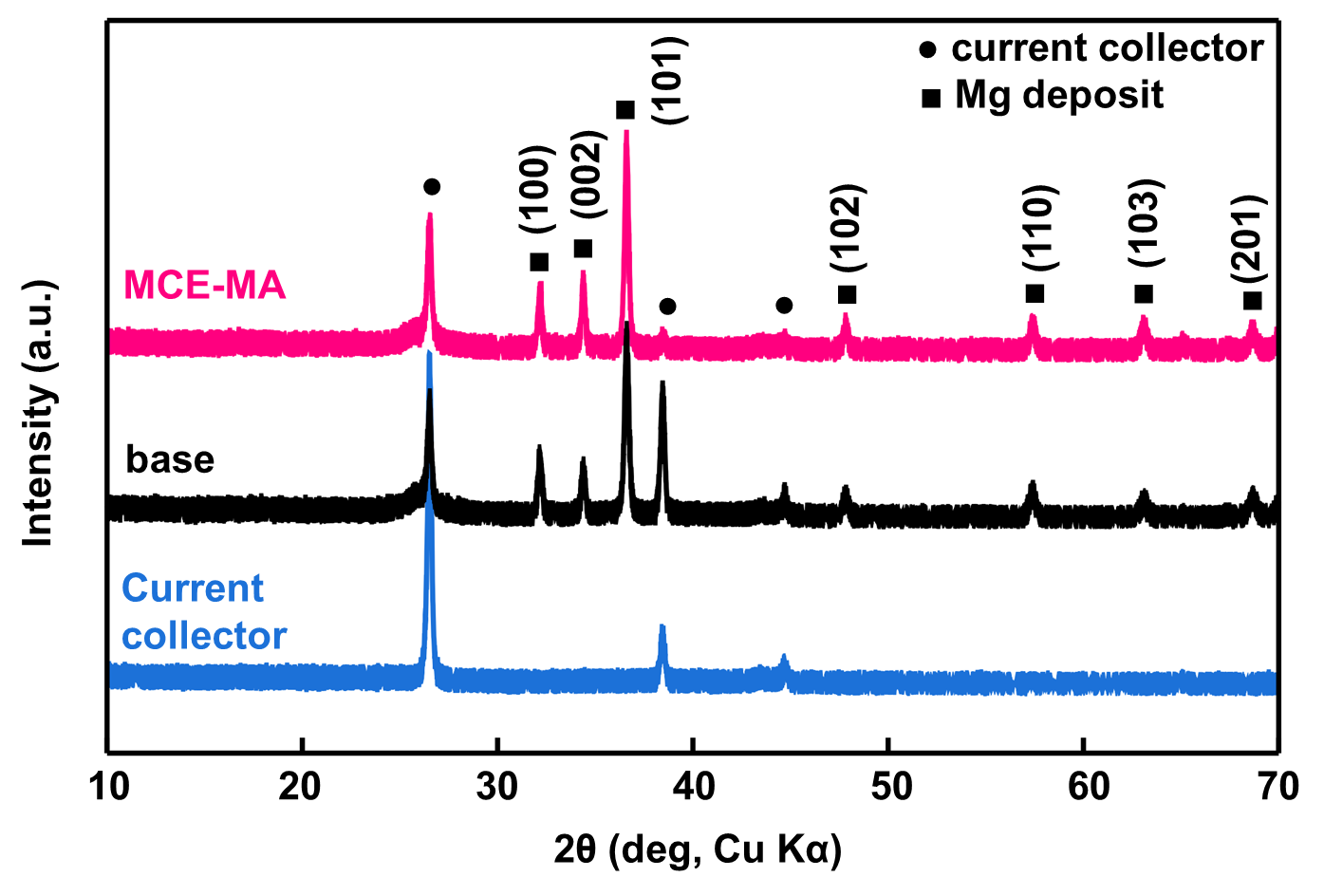


Figure S14. XRD profile comparison of Mg deposit on graphite-coated aluminum foil as current collector using base electrolyte and MCE-MA.

**References**

[1] Z. Song, Z. Zhang, A. Du, S. Dong, G. Li, G. Cui, “Insights into interfacial speciation and deposition morphology evolution at Mg-electrolyte interfaces under practical conditions” *J. Energy Chem.* **2020**, *48*, 299–307.

[2] Z. Li, T. Diemant, Z. Meng, Y. Xiu, A. Reupert, L. Wang, M. Fichtner, Z. Zhao-Karger, “Establishing a Stable Anode–Electrolyte Interface in Mg Batteries by Electrolyte Additive” *ACS Appl. Mater. Interfaces* **2021**, *13*, 33123–33132.

[3] E. Winter, T. J. Schmidt, S. Trabesinger, “Identifying Pitfalls in Lithium Metal Battery Characterization” *Batter. Supercaps* **2022**, *5*, DOI 10.1002/batt.202100145.
